# Invertebrates at the Land‐Sea Interface in a Climate Change World: Heatwaves, Floods, Fire and Ice

**DOI:** 10.1111/gcb.71096

**Published:** 2026-09-07

**Authors:** Maria Byrne, Pauline M. Ross

**Affiliations:** ^1^ Marine Invertebrate Futures Group, School of Life and Environmental Sciences The University of Sydney Sydney New South Wales Australia

**Keywords:** atmospheric heatwaves, coastal wildfires, extreme climatic events, flooding‐hyposalinity, marine heatwaves, marine invertebrates

## Abstract

With their location at the land‐sea edge, coastal marine invertebrates withstand marked changes in water and air temperatures and abrupt salinity changes through tidal cycles. In a climate‐changing world, these stressors are being magnified by the increased intensity and emergence of extreme climatic events (ECEs). Diverse habitat‐dependent communities on rocky shores, mudflats, mangroves and saltmarsh are at the forefront of ECEs, which can co‐occur and interact with other anthropogenic stressors. This review synthesises information on the vulnerabilities of coastal marine invertebrates living at the interface between marine and terrestrial environments to marine and atmospheric heatwaves, fire, flooding, hyposalinity and changes in intertidal ice cover. The impacts of ECEs on bivalves are investigated as these are ecologically and economically important keystone species. We synthesise impacts of extreme heat stress on these molluscs as a case study. Temperature and salinity extremes are particularly damaging to coastal invertebrate communities if they coincide with low tide cycles. As adaptation to environmental variability is an inherent feature of the evolution in the intertidal, it will be important to understand tolerance levels with respect to ECEs, as this varies between species and across tidal cycles. It is also important to understand the capacity to build climate resilience through acclimatisation, adaptation, plasticity and the presence of refuge habitats, as well as identify the potential for species' redundancy with respect to the provision of key ecosystem services.

## Graphical abstract

Diverse communities of coastal marine invertebrates living on rocky shores, mudflats, mangroves and saltmarsh occur at the interface between marine and terrestrial environments, where they are vulnerable to the combined impacts of extreme climatic events (ECEs) such as marine and atmospheric heatwaves, fire, flooding, hyposalinity and changes in intertidal ice cover. At the land‐sea edge, these ECE can co‐occur and interact with other stressors such as pollution and urbanisation. Heat and salinity extremes are particularly damaging to coastal invertebrate communities if they coincide with low‐tide cycles. This review synthesises information on the vulnerabilities of coastal marine invertebrates to the impacts of ECEs. We synthesise the impacts of extreme heat stress on oysters and mussels as a case study, as these are ecologically and economically important species. Understanding the impacts of ECEs on coastal invertebrates is key, as these organisms are ecologically important food resources for higher trophic levels (e.g., shorebirds, fishes) and provide many vital ecosystem services. This knowledge is essential to manage coastal marine resources on which human communities depend.

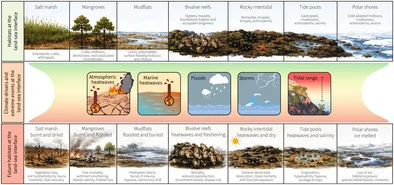

## 1 Introduction

**FIGURE 1:**
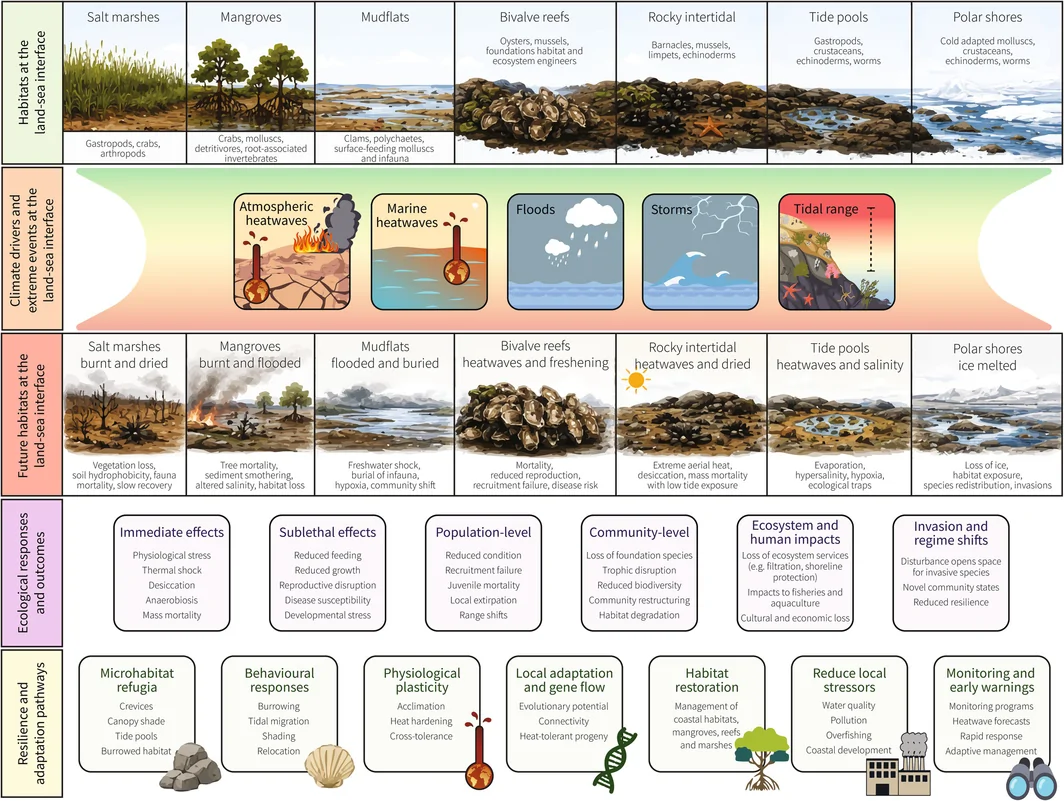
Extreme climatic events impacting coastal marine habitats, stressor impacts, ecological responses and potential resilience mechanisms.

Marine ecosystems along the coastal rim at the land‐sea edge are faced with a combined suite of stressors from marine and terrestrial realms, including extreme climatic events (ECEs), marine and atmospheric heatwaves, coastal wildfires, flood‐driven hyposalinity and changes in ice cover. In a climate‐changing world, these stressors are being magnified, with diverse habitat‐dependent communities on rocky shores, mudflats, mangroves and saltmarsh at the forefront of these perturbations (Figure 1).

Marine and atmospheric temperatures are increasing due to anthropogenic CO_2_ emissions, and this is being exacerbated by the increased intensity and frequency of heatwaves (Stillman 2019; *IPCC* 2023; Smith et al. 2023; Wernberg et al. 2024; Stillman et al. 2025). These events can drive sudden and severe degradation of coastal ecosystems. Heatwaves are contemporary recurring emergencies impacting inhabitants of the land‐sea interface and will continue to do so for the foreseeable future.

**FIGURE 2:**
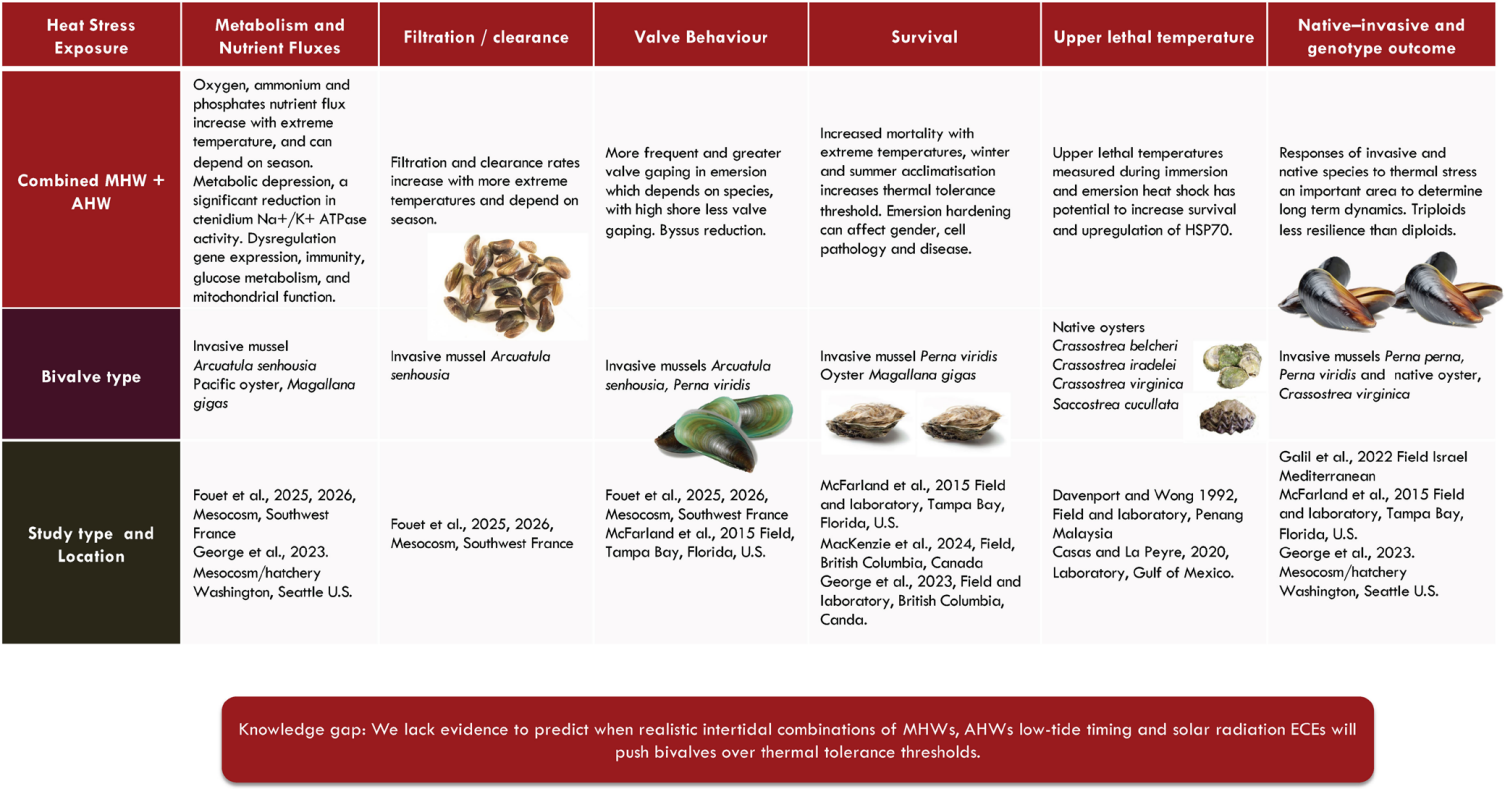
Impacts of combined MHW and AHW on metabolism and nutrient flux, filtration and clearance rate, valve behaviour, including clapping, survival, upper lethal temperatures, and responses of native and invasive mussels and oysters in the field, laboratory, mesocosm and hatchery experiments, and locations investigated around the world. Images from: *Arcuatula senhousia* https://www.seanature.co.uk/arcuatula_senhousia.html. *Crassostrea cucullata* Atlas of Living Australia https://bie.ala.org.au/species/https://biodiversity.org.au/afd/taxa/72fe1097‐f7c8‐4769‐80ca‐51244742863f. *Crassostrea virginica* https://www.seacoreseafood.com/product/st‐peters‐oysters. *Magallana gigas* https://www.spend‐in.com/food/ostra2. *Perna viridis* https://nimpis.marinepests.gov.au/species/species/35. *Perna perna* https://www.pngegg.com/en/search?q=Perna+viridis

Intertidal marine invertebrates live in a multistressor world where climate and other anthropogenic stressors such as coastal pollution and urbanisation coincide (Figure 1). In the Anthropocene, the concerns are that temperatures are increasing so that they are now reaching levels inconsistent with life (Stillman 2019). Our understanding of how combined marine and atmospheric heatwaves impact coastal marine communities and ecosystems is limited. This review synthesises information on the vulnerabilities of coastal marine invertebrates to increasing ECEs, with a focus on heatwaves and associated changes in fire regimes, as well as to rainfall‐hyposaline events and changes in intertidal ice cover (Figure 1). As bivalves (e.g., oysters and mussels) provide keystone ecosystem services along the coastal margin, supporting a diversity of species, we assess studies of the impacts of marine and atmospheric heatwaves on these molluscs as a focal taxon (Figure 2). As other climate stressors, including ocean acidification and poleward range extensions of marine species due to warming, are synthesised in several reviews (e.g., Pecl et al. 2017; Byrne and Fitzer 2019) these are not covered here. Understanding the impacts of ECEs on coastal invertebrates is key, as these organisms are ecologically important food resources for higher trophic levels (e.g., shorebirds, fishes) and provide vital ecosystem services. This knowledge is essential to manage coastal marine resources on which human communities depend.

## 2 Marine and Atmospheric Heatwaves at the Land‐Sea Margin

The duration and intensity of combined marine and atmospheric heatwaves are important determinant of their impact on coastal biodiversity (Stillman et al. 2025). Marine heatwaves (MHWs) may last for weeks or months and over broad spatial extents and so can have extensive negative impacts on marine organisms and ecosystems (Hobday et al. 2016; *IPCC* 2023; Smith et al. 2023; Wernberg et al. 2024). In parallel, the incidence of atmospheric heatwaves (AHWs) is increasing. Due to tightly coupled ocean‐atmospheric feedback loops, marine and atmospheric heatwaves often co‐occur with dire impacts on biodiversity (Cook et al. 2022; Stillman et al. 2025). This feedback is also driving a decline in sea‐land breezes and associated cooling of the coastal margin (Xiao et al. 2026). It is expected that land and sea heat anomalies will become more intense and frequent (*IPCC* 2023) and are likely to act in a synergistic manner to form compound events.

Marine ectotherms are directly impacted by habitat warming due to the strong influence of temperature on physiological processes, energy budgets and overall organism functioning. Increasing temperatures have a pervasive stimulatory effect on metabolism until lethal levels are reached in association with hypoxia/anaerobiosis and cellular dysfunction (Hofmann and Todgham 2010; Stillman 2019). Many intertidal species are sessile and are particularly vulnerable to simultaneous land‐sea warming as they cannot move away from danger.

Adaptation to environmental variability is an inherent feature of evolution to life in the intertidal. Temperatures can pivot markedly between high temperatures at low tide and marked cooling with tidal emersion (Przeslawski and Davis 2007; Lathlean et al. 2011; Wolfe et al. 2020). The timing of tides and local weather patterns can be crucial with respect to protection—relief from stress on one hand or increased risk on the other (Helmuth et al. 2006; Leeuwis and Gamperl 2022). Thermal extremes are particularly damaging if they coincide with low‐tide cycles, as these events can drive physiological shifts in energetics and local extirpation (Helmuth et al. 2006; Raymond et al. 2022). Extreme marine and atmospheric heatwaves as single or combined stressors have caused mass mortality of rocky shore barnacles, mussels, and oysters and mudflat bivalves (Harley 2008; Seuront et al. 2019; Scanes et al. 2020; Raymond et al. 2022; Hesketh and Harley 2023).

Tidepools provide refuge for intertidal species from increased temperature and desiccation at low tide, but when they become hypersaline, hypoxic and warm beyond tolerance levels during AHW, mortality occurs (Firth and Williams 2009; Vinagre et al. 2018). This tidepool‐ecological trap appears to be worse for tropical than temperate species (Vinagre et al. 2018). In association with their more thermally stable habitat, tropical species have smaller thermal safety margins (TSMs) and live closer to their heat‐tolerance limits than temperate species, which experience wider, more variable temperatures and have broader TSMs and thereby greater thermal tolerance (Vinagre et al. 2018, 2019; Pinsky et al. 2019; Stillman et al. 2025).

Intermittent exposure to elevated temperatures over consecutive low tides during AHW can assist in acclimatisation to extreme heat in some species, but not others (Jones et al. 2009; Vinagre et al. 2018) and this can vary with life history stage. For example, intertidal sea stars ( *Parvulastra exigua* ) are resilient to MHW as adults, but negative carry‐over effects were evident for offspring (Balogh and Byrne 2020). For the keystone predator *Pisaster ochraceus* , AHW may limit foraging, but survival is facilitated by thermoregulatory strategies (Princebourde et al. 2009). In the sea urchin *Heliocidaris erythrogramma*, MHW conditions increased metabolism but without increasing feeding, resulting in an energy deficit with lasting physiological effects, disease, mortality, and negative carry‐over effects for offspring (Minuti et al. 2021). For oysters, prior exposure to heat shock may increase survival at elevated temperatures (Pereira et al. 2020; Georgoulis et al. 2023).

It has been suggested that although intertidal species have inbuilt genetic and phenotypic plasticity to be resilient to thermal stress, many of these species live close to their upper thermal limits (Somero 2002; Wethey 2002; Blewett et al. 2022). Intertidal animals can tolerate wide‐ranging temperatures (Somero 2002), but as heatwaves become more common and of longer duration, they may have to compromise their fitness or succumb to the accumulated effects of heat exposure.

## 3 Bivalves in the Heat

Bivalve reefs worldwide form complex three‐dimensional biogenic habitats that modify environmental conditions and provide habitat and food for a vast biodiversity. Thus, the impacts of heatwaves on bivalve reefs extend well beyond the bivalves themselves in altering ecological communities and functions (Wernberg et al. 2024). When AHWs coincide with low tides during the day, mortality varies with respect to shore level, orientation to solar radiation, life history stage, and whether bivalves occur singly or in aggregations (Wernberg et al. 2024). Clumped individuals may experience lower thermal stress through greater water retention, whereas individuals on equatorward‐facing surfaces experience more intense heating (Harley 2008; McAfee et al. 2017). Bivalves that live lower on the shore or deeper in the mud are less affected by AHWs than higher‐shore and surface‐living species (Raymond et al. 2022). During emersion, high temperatures and AHW conditions contributed to mass mortality of Pacific oysters (*Magallana gigas*) in South Korea (Heo et al. 2023). Some species exhibit tolerance to land‐sea heatwaves, although they may experience sublethal impacts on growth, feeding, and reproduction (Vázquez et al. 2021; Román et al. 2025). While heatwaves may occur for a short period of a bivalve's life, the impacts can be long lasting if they occur in concert with reproduction (Stillman 2019).

While the physiological effects of aerial exposure on bivalves have long been recognised (e.g., Bayne et al. 1976; Helmuth et al. 2006; Bayne 2017), less is known about the responses of intertidal bivalves to the effects of combined seawater and aerial temperatures and other factors such as alterations to primary productivity and food availability (Schneider et al. 2010). Interest is now focused on understanding how extreme heatwaves impact intertidal bivalves (Figure 2). Extreme AHW conditions exceeding 40°C–50°C for 5 or more days caused mass mortality of mussels and oysters across Europe, North America and Israel (Seuront et al. 2019; Galil et al. 2022; Miner et al. 2025). Juvenile, invasive *Perna perna* were particularly affected, with near‐complete disappearance (Galil et al. 2022), likely due to impaired byssal thread production at temperatures above 32°C (Rajagopal et al. 1995). In Europe, *M. gigas* replaced native mussels and oysters following consecutive warm summers (Diederich 2006). Extreme heatwaves cause large ecological and economic impacts, highlighting the need to determine factors affecting emersion time, communities most at risk and species‐dependent recovery processes, which create species cascades (Miner et al. 2025).

To determine current levels of research and understanding on the responses of intertidal bivalves to MHW, AWH and their combined effects, we conducted a Preferred Reporting Items for Systematic Reviews and Meta‐Analyses (PRISMA) (See Supporting Information). We identified 1028 studies for title and abstract screening, with 390 studies on heat stress, but not extreme heatwaves. With further screening, 193 were identified as relevant (MHW, 140; AHW, 44; MHW + AHW, 9). The combined effects of AHWs and MHWs were experimentally investigated in nine species; five oyster species (Casas and La Peyre 2020; George et al. 2023; Davenport and Wong 1992; Mackenzie et al. 2024) and four mussels (McFarland et al. 2015; Galil et al. 2022; Fouet et al. 2025, 2026). Responses of six species (four mussels, two clams) to thermal stress were modelled (Ma et al. 2024). No studies were found examining the combined effects of MHW and AWH on clams. In general, there remains a lack of studies examining multiple life stages and mechanisms that confer thermal resilience.

Many studies on the combined effects of AHW and MHW are on invasive species (Figure 2). For example, McFarland et al. (2015) found greater heat stress tolerance in the native oyster *C. virginica* compared with the invasive mussel *Perna viridis* while Fouet et al. (2025, 2026) exposed the invasive mussel *Arcuatula senhousia* to simulated MHW and AHW and found altered nutrient and oxygen fluxes at the sediment–water interface that may enhance their invasive capacity. George et al. (2023) found greater mortality, metabolic depression, and altered gene expression associated with stress, immunity, and metabolism in *M. gigas* when MHW and AHW were a combined stress compared with MHW alone, with greater impacts on the survival of triploids than diploids. Mackenzie et al. (2024) found greater survival of *M. gigas* when immersed after exposure to an AHW.

The greater research focus on invasive rather than native species creates a gap in understanding of how extreme heatwaves and climate change will impact ecosystems and the ecosystem services bivalves provide. Species that are of aquacultural importance and have an invasion risk often receive greater attention because of economic and management incentives.

## 4 Fire and Ice

**FIGURE 3:**
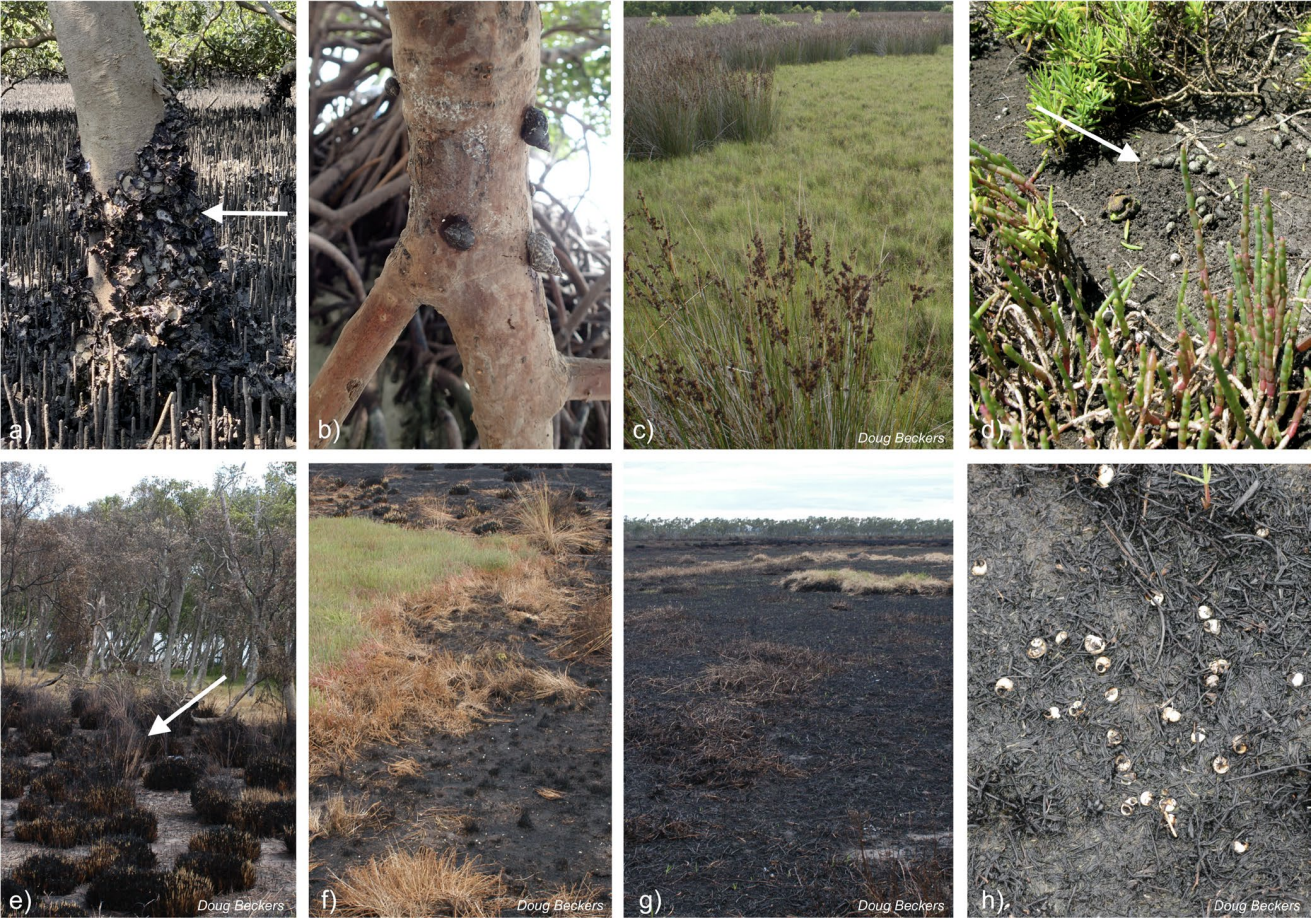
Unburnt (top) and burnt (bottom) saltmarsh and mangrove habitats in New South Wales (NSW) (a, c–h) and Queensland (b), Australia. (a) Sydney rock oysters, *Saccostrea glomerata* (arrow) on the trunk of the grey mangrove *Avicennia marina* . (b) Gastropods, *Cerithidea* spp. on the red mangrove *Rhizophora stylosa* (c) *Sporobolus virginicus* saltmarsh surrounded by the taller rush *Juncus krauss*ii at Ash Island, Kooragang Hunter Wetland Ramsar site. (d) Gastropods, *Ophicardelus* spp. and *Phallomedusa solida* (arrows) among *Salicornia quinqueflora* and *Suaeda australis*. (e) Burnt grey mangrove in the background with the invasive *Juncus acutus* in the foreground (arrows). (f), (g) Burnt *Sporobolus virginicus* and *Salicornia quinqueflora* saltmarsh (h). Dead gastropods *Ophicardelus* spp. and *P. solida* at Ash Island. All photos are by the authors except for figures c, e, f, and g, courtesy of Doug Beckers, NSW Department of Climate Change, Energy, the Environment and Water.

Droughts and climate change‐driven warming, together with lunar cycle modulation of maximum tide height, can cause drying of vegetation at the land's edge, as seen in the vast die‐off of mangroves (Saintilan et al. 2022). Vegetation drying can lead to wildfires (Ross and Adam 2013; Duke et al. 2017; Ross et al. 2019; Bracewell et al. 2023; Glasby et al. 2023). Recent coastal fires have presented an unprecedented stressor to marine invertebrates, with destructive incineration of key habitats such as saltmarsh and mangroves and mass loss of habitat‐dependent species (Figure 3). Coastal fires can cause long‐term change, as many of the impacted habitats have poor recovery prospects, with burnt mangroves standing as dead trees 2 years later (Glasby et al. 2023). Fire followed by rain can inundate coastal species with sediment, ash and other debris that often have associated toxic contaminants (Bracewell et al. 2023; Bragg et al. 2026). Changes to sediment communities were observed following wildfires (Bracewell et al. 2023).

Wildfires are most frequently considered as catastrophic terrestrial events, together with the impacts of post‐fire rainfall runoff on aquatic catchments and biota (Review, Gomez Isaza et al. 2021). Very little research has been done on the impacts of fire on coastal ecosystems. Only one study from eastern Australia explicitly investigated the impact of fire on saltmarsh gastropods and found differences in their abundance between burnt and unburnt sites 2 years post‐fire (Ross et al. 2019). A recent study of the impacts of a coastal wildfire (California, USA) attributed a 21% loss of rocky intertidal habitat and mass mortality of invertebrates, including a 59.6% loss of the endangered abalone (*Haliotis cracerodii*) to burial (3.5 m of sediment) in post‐fire debris flows (Bragg et al. 2026).

In the Arctic, extreme atmospheric and marine warming is causing a reduction in and changes in the timing of ice cover (Berge et al. 2005; Grebmeier 2012; Gou et al. 2025). Ice‐free intertidal habitat enabled the reappearance of *Mytilus edulis* after a 1000‐year absence in the Baltic (Berge et al. 2005). As ice cover decreases and the environment warms, intertidal communities are changing in parallel with system‐wide reorganisation of Arctic ecosystems (Grebmeier 2012; Kortsch et al. 2012; Byrne and Lamare 2024). In the Canadian Arctic, reduction in sea ice and thereby intertidal ice scour due to warming is driving change in intertidal communities, as seen in the increase in barnacles and algae (Scrosati et al. 2025).

## 5 Coastal Storms and Flooding—Hyposalinity Events

Climate change is modifying rainfall patterns and increasing the intensity of storms (cyclones/typhoons/hurricanes), resulting in more frequent coastal flooding and elevated river discharge (*IPCC* 2023; Malan et al. 2024). These ECEs increase shear stress on the benthos, causing physical removal and damage to intertidal species, as well as inundation by mobilised sediment, resulting in mass loss of species and changes to community assemblages (Engle et al. 2009; Taillie et al. 2020; Shi et al. 2021). After Hurricane Katrina (USA), there was a significant reduction in the biodiversity of soft sediment communities (Engle et al. 2009). Similarly, Typhoon Lionrock, despite being 1400 km away from the coast (Jiangsu, China), increased bed shear, causing rapid erosion of intertidal mudflats and mass species loss, including a 50% reduction of the important clam *Meretrix meretrix* (Shi et al. 2021). In typhoon‐impacted mangrove habitat where 100% mortality of the trees occurred, ecologically important crab populations were able to persist, but with a change in species composition and a reduction in body size in areas where the canopy was removed (Diele et al. 2013).

Intense rain accompanying storms can cause a sudden drop in salinity due to freshwater influx. These conditions are often lethal to marine species (Webb et al. 2025; Waller et al. 2026). The impacts are greatest when intense runoff coincides with low tide, resulting in compound stressors. Some coastal species have a broad salinity tolerance, reducing the impacts of freshening. For instance, *M. gigas* can tolerate a salinity range of 5–40 ppt (Meng et al. 2013). For other species, flash flooding creates acute hyposalinity, causing mass mortality (Webb et al. 2025; Waller et al. 2026). Low salinity stress can also cause sublethal effects, such as increased susceptibility to disease, and reduced feeding and reproduction (Gillanders and Kingsford 2002; Domínguez et al. 2020).

## 6 Refugia and Protection in the Face of Change

The diverse geomorphology, oceanographic regimes and structural complexity of coastal habitats present a myriad of environmental variations which influence organism stress exposure levels. These variations include differences in aspect, from full solar exposure to full shade, low complexity rock platforms to intricate tree root systems, as well as differences in sediment types. The nature of the local environment creates a mosaic of microclimates which determines the severity of stress and levels of body heating. This is an important factor to understand at the level of the organism (Helmuth et al. 2010).

Species that are thermal specialists and which have narrow distributions appear more prone to local extirpation in response to warming (Grinder and Wiens 2022). Poor success or local extirpation of native species due to climate stress can provide an opportunity for invasive species to become established (Sorte, Williams, and Carlton 2010; Papacostas et al. 2017), although natives can have greater thermal tolerance than invasives, with other stressors determining outcomes (McFarland et al. 2015).

Widely distributed species can occupy a range of environmentally heterogeneous habitats, with populations in more protected habitats providing some redundancy. Populations in refugia habitats less impacted by ECEs, where native taxa can maintain viability, can be the source of propagules to reseed impacted populations. Such climate change refugia are important for marine conservation (see Rilov et al. 2026).

## 7 Potential for Acclimatisation and Adaptation

If coastal species are to persist in the face of changing climate and ECEs, they will need to be resilient, to respond and recover from stress. There are two ways that organisms can build resilience: acclimatisation or adaptation. Acclimatisation involves phenotypic changes in physiology, morphology, or behaviour which improve the response of an organism in the face of stress without altering their genotype (Ross et al. 2016, 2023). Stress‐induced adaptive evolution may also occur, although this is mostly reported for vertebrates (Mojica and Kültz 2022). Evolutionary adaptation is typically a long‐term, multigenerational selective process and may be too slow to keep up with the pressures wrought by ECEs (Byrne et al. 2020).

With successive exposure to heatwaves, intertidal species can acclimatise to have increased heat tolerance (heat‐hardened/heat‐prepared) as shown for gastropods (e.g., *Nucella, Littorina*) or become more sensitive, as shown for a bivalve ( *Mytilus edulis* ) (decreased heat tolerance/heat softening) (Jones et al. 2009; Woodin et al. 2013; Truebano et al. 2018; Seuront et al. 2019). For *Saccostrea glomerata* heat hardening conveyed resilience to stress quickly, both within and across generations (Scanes et al. 2020). There can also be negative carryover effects on the progeny of parents that experience MHW (Balogh and Byrne 2020; Minuti et al. 2021). Animals adapted to warm environments tend to have the least plasticity in their heat tolerance, indicating that the ability of plasticity/acclimatisation to keep pace with increasing temperature is limited (Gunderson et al. 2016; Stillman 2019). Thus, tropical species may be more vulnerable to extreme warming than temperate species (Vinagre et al. 2018; Stillman et al. 2025).

Importantly, as most marine invertebrates have planktonic larval and benthic adult phases, the integration of climate stress resilience/acclimatisation and vulnerabilities across life history stages is key to species persistence in a changing ocean (Byrne 2011; Byrne et al. 2018). Many life history bottlenecks can influence species' success with respect to environmental conditions such as sea temperature for larvae and sea and air temperature for adults. Reduced reproductive output and fitness of benthic adults or success of early juveniles due to MHW can result in recruitment failure, compromising population persistence (Shanks et al. 2020; Correia‐Martins et al. 2022). For many marine invertebrates, early stages are most sensitive to stress (Przeslawski et al. 2015; Truebano et al. 2018; Pandori and Sorte 2019; Byrne and Lamare 2024). With respect to species range change in a warming climate, the thermal biology of dispersive larvae can be most important (Byrne et al. 2022). Ocean warming can favour the propagules of invasive/pest species which have high plasticity and tolerance across a range of environmental stressors (Sorte, Fuller, and Bracken 2010; Thomson et al. 2025). In many coastal habitats, invasive species are emerging as winners in the climate change stakes, as they have high tolerance to a range of stresses across their life cycle (Papacostas et al. 2017).

Finally, while not a focus of this review, it is important to mention that sea level rise is an existential global threat to communities that have evolved to live in the intertidal (Seeger and Minderhoud 2026). This ongoing process is causing coastal squeeze, drowning of many habitats, and disappearance of habitat‐dependent species (Ross and Adam 2013).

## 8 Conclusions, Knowledge Gaps & Management Strategies

Intertidal marine communities are at the front line of global change, in experiencing ECEs, and can serve as a bellwether of the impacts of these events, but knowledge gaps need to be addressed to inform management strategies.

1. Design ECE‐responsive monitoring protocols: Adaptive monitoring of coastal communities in parallel with the suite of environmental parameters/stressors is needed to understand natural variability with respect to immersion‐emersion tidal cycles. This is essential to identify anomalous changes and tease out the impacts of ECEs, to understand vulnerabilities and stress tolerance, and to predict/model future impacts. This must be biologically relevant to understand organism‐level variation.

2. Use data from real‐world conditions to inform controlled laboratory and mesocosm experiments: This is needed to tease out the interactive impacts of background abiotic flux and ECEs, to identify mechanisms of vulnerability/resilience and species' tolerance limits through physiological studies, and to understand responses of native and non‐native species to predict impacts on ecosystem services and economic risks and inform management practices.

3. Understanding the potential for contrasting responses to combined MHW ± AHW: (e.g., heat hardening vs. heat softening) for ecologically influential species such as bivalves is a key knowledge gap.

4. Monitor populations post‐ECEs as these can have sublethal to lethal impacts: This is needed to determine recovery, long‐term legacy effects, and the potential for species' redundancy with respect to key ecosystem services.

5. Prioritise climate refugia in conservation frameworks: Identify areas and habitats where climate change impacts will remain low to improve the resilience of species and ecosystems (see Rilov et al. 2026).

## Author Contributions

**Maria Byrne:** conceptualization, visualization, writing – review and editing, writing – original draft, investigation. **Pauline M. Ross:** investigation, funding acquisition, writing – review and editing, writing – original draft, visualization, methodology.

## Conflicts of Interest

The authors declare no conflicts of interest.

## Supporting information


**Data S1:** Supporting Information.

## Data Availability

No new data were generated for this manuscript. All details on the terms used for the systematic review are in the supporting information.
