# Supplementary material for: Invertebrates at the Land‐Sea Interface in a Climate Change World: Heatwaves, Floods, Fire and Ice
Source: Glob Chang Biol. 2026 Sep 7;32(9):e71096. doi: 10.1111/gcb.71096 (PMC13548343; doi:10.1111/gcb.71096)
Supplement: Supplementary file 1 [file GCB-32-e71096-s001.docx]

**Supplementary Material**

**Full description of Systematic Review**

These search terms below were inputted into Scopus database on the 18^th^ of May 2026. **1028** publications were pooled from these search terms.

Full Search Term

**[**"Bivalve" OR "Intertidal bivalve" OR "*Oyster" OR "*Mussel" OR "*Clam" OR "*Cockle" OR "*Scallop" OR "*Mytilus edulis*" OR "Blue mussel" OR "*Pinctada maxima*" OR "*Ostrea angasi*" OR "Geukensia demissa" OR "*Saccostrea glomerata*" OR "*Mytilus californianus*" OR "*Ruditapes philippinarum*" OR "*Perna canaliculus*" OR "*Crassostrea gigas*" OR "*Xenostrobus securis*" OR "*Mytilus galloprovincialis*" OR "*Mytella strigata*" OR "*Macoma balthica*" OR "*Corbicula fluminea*" OR "Pacific rock oyster" OR " Sydney rock oyster" OR “Flat oyster” OR “ Green-lipped mussels” AND "Atmospheric heatwave" OR "AHW" OR “Aerial exposure” OR “byssus” OR “heatwave” OR “MHW” OR “marine heatwave” OR “thermal stress” OR “desiccation”**]**

It is estimated that from these **1028** publications that **252** were in relation to oxidative stress and desiccation leaving **776** papers relevant to heat induced stress. A total of **390** of the publications were relevant to heat stress but not relevant to extreme events such as heatwaves. After screening twice for relevance in terms of bivalves studied, experiments relevant to the impact of heatwaves on intertidal bivalves rather than reviews **193** studies were included. . Of these studies **140** studies were found to be solely relevant to MHWs, **44** studies were screened to be relevant to AHWs and **9** studies were found to assess combined effects, however, these took many forms and were rarely phrased as testing both AHW and MHW.

**Referenc3w**

Acquafredda, M. P., Calvo, L., De Luca, M., & Munroe, D. (2026). Evaluating farmed Atlantic surfclam (*Spisula solidissima*) grow-out performance across three New Jersey bays. *Journal of Shellfish Research*, **44**(3), 387-403. https//doi.org/10.2983/035.044.0306

Ait Chattou, E. M., Abounahel, N., Kassila, J., Ouagajjou, Y., Abouhala, A., Idhalla, M., & Moukrim, A. (2019). Differential growth of the brown mussel, *Perna perna* (Linnaeus, 1758), in longline and pole cultures in Dakhla Bay (SW Morocco, Atlantic Ocean). *Aquaculture Research*, **50**(3), 736-747. https//doi.org/10.1111/are.13927

Aldridge, D. W., & Payne, B. S. (2001). Effects of temperature and aerial exposure on the BOD of waste zebra mussels removed from navigational locks. *Water Research*, **35**(12), 2970-2974. https//doi.org/10.1016/S0043-1354(01)00006-9

Ali, F., & Nakamura, K. (2000). Metabolic characteristics of the Japanese clam *Ruditapes philippinarum* (Adams and Reeve) during aerial exposure. *Aquaculture Research*, **31**(2), 157-165. https//doi.org/10.1046/j.1365-2109.2000.00402.x

Almeida, E. A., Dias Bainy, A. C., Dafre, A. L., Gomes, O. F., Medeiros, M. H. G., & Di Mascio, P. (2005). Oxidative stress in digestive gland and gill of the brown mussel (*Perna perna*) exposed to air and re-submersed. *Journal of Experimental Marine Biology and Ecology*, **318**(1), 21-30. https//doi.org/10.1016/j.jembe.2004.12.007

Alter, K., Jacobs, P., Delre, A., Rasch, B., Philippart, C. J. M., & Peck, M. A. (2024). Oyster larvae used for ecosystem restoration benefit from increased thermal fluctuation. *Marine Pollution Bulletin*, **198**. https//doi.org/10.1016/j.marpolbul.2023.115750

Altieri, A. H. (2006). Inducible variation in hypoxia tolerance across the intertidal-subtidal distribution of the blue mussel *Mytilus edulis*. *Marine Ecology Progress Series*, **325**, 295-300. https//doi.org/https//doi.org/10.3354/meps325295

Amorim, V. E., Gonçalves, O., Capela, R., Fernández-Boo, S., Oliveira, M., Dolbeth, M., Arenas, F., & Cardoso, P. G. (2020). Immunological and oxidative stress responses of the bivalve *Scrobicularia plana* to distinct patterns of heatwaves. *Fish and Shellfish Immunology*, **106**, 1067-1077. https//doi.org/10.1016/j.fsi.2020.09.024

Andréfouët, S., Bouchet, N., Bruyère, O., Basset, C., Bionaz, O., Bourgeois, B., Le Gendre, R., Liao, V., Marcellino, R., Trophime, T., Teaniniuraitemoana, V., & Lo-Yat, A. (2026). Decline and status of natural pearl oyster (*Pinctada margaritifera*) stocks in two French Polynesia black pearl farming lagoons. *Marine Pollution Bulletin*, **222**. https//doi.org/10.1016/j.marpolbul.2025.118852

Arduini, D., Portacci, G., Giangrande, A., Acquaviva, M. I., Borghese, J., Calabrese, C., Giandomenico, S., Quarta, E., & Stabili, L. (2023). Growth performance of *Mytilus galloprovincialis* Lamarck, 1819 under an innovative integrated multi-trophic aquaculture system (IMTA) in the Mar Grande of Taranto (Mediterranean Sea, Italy). *Water (Switzerland)*, **15**(10). https//doi.org/10.3390/w15101922

Azizan, A., Venter, L., Jansen van Rensburg, P. J., Ericson, J. A., Ragg, N. L. C., & Alfaro, A. C. (2023). Metabolite changes of *Perna canaliculus* following a laboratory marine heatwave exposure: Insights from metabolomic analyses. *Metabolites*, **13**(7). https//doi.org/10.3390/metabo13070815

Babarro, J. M. F., & De Zwaan, A. (2008). Anaerobic survival potential of four bivalves from different habitats. A comparative survey. *Comparative Biochemistry and Physiology - A Molecular and Integrative Physiology*, **151**(1), 108-113. https//doi.org/10.1016/j.cbpa.2008.06.006

Barbosa, H., Soares, A. M. V. M., Pereira, E., & Freitas, R. (2023). Are the consequences of lithium in marine clams enhanced by climate change? *Environmental Pollution*, **326**. https//doi.org/10.1016/j.envpol.2023.121416

Bartsch, M. R., Waller, D. L., Cope, W. G., & Gutreuter, S. (2000). Emersion and thermal tolerances of three species of unionid mussels: Survival and behavioral effects. *Journal of Shellfish Research*, **19**(1), 233-240. https//doi.org/https//doi.org/

Bates, H., Pierce, M., & Benter, A. (2021). Real-time environmental monitoring for aquaculture using a lorawan-based iot sensor network. Sensors, **21**(23). https//doi.org/10.3390/s21237963

Beaudreau, N., Page, T. M., Drolet, D., McKindsey, C. W., Howland, K. L., & Calosi, P. (2024). Using a metabolomics approach to investigate the sensitivity of a potential Arctic-invader and its Arctic sister-species to marine heatwaves and traditional harvesting disturbances. *Science of the Total Environment*, **917**. https//doi.org/10.1016/j.scitotenv.2024.170167

Beck, E. L., Ruesink, J., Troyer, S., & Behrens, M. (2024). Wild populations of Pacific oysters (*Magallana gigas*) emerge during the blob heatwave in south Puget Sound, Washington USA. *Frontiers in Marine Science*, **11**. https//doi.org/10.3389/fmars.2024.1292062

Belgrad, B. A., Cooper, N., & Smee, D. L. (2026). Structured habitats provide thermal refuges and mitigate effects of heatwaves. Biological Conservation, 313. https//doi.org/10.1016/j.biocon.2025.111634

Benjamin, E. D., Jeffs, A., Handley, S. J., Toone, T. A., & Hillman, J. R. (2023). Determining restoration potential by transplanting mussels of different size classes over a range of aerial exposures. *Marine Ecology Progress Series*, **713**, 71-81. https//doi.org/10.3354/meps14337

Benjamin, E. D., Toone, T. A., Hillman, J. R., Handley, S. J., & Jeffs, A. (2024). Aerial exposure and critical temperatures limit the survival of restored intertidal mussels*. Restoration Ecology*, **32**(4). https//doi.org/10.1111/rec.14105

Bertolini, C., Glaser, D., Canu, M., & Pastres, R. (2023). Coupling habitat-specific temperature scenarios with tolerance landscape to predict the impacts of climate change on farmed bivalves. *Marine Environmental Research*, **188**. https//doi.org/10.1016/j.marenvres.2023.106038

Bertolini, C., & Pastres, R. (2021). Tolerance landscapes can be used to predict species-specific responses to climate change beyond the marine heatwave concept: Using tolerance landscape models for an ecologically meaningful classification of extreme climate events. *Estuarine, Coastal and Shelf Science*, **252**. https//doi.org/10.1016/j.ecss.2021.107284

Borrero, F. J. (1987). Tidal height and gametogenesis: reproductive variation among populations of *Geukensia demissa*. *Biological* Bulletin, **173**(1), 160-168. https//doi.org/10.2307/1541869

Bost, M. C., Rodriguez, A. B., Ridge, J. T., Miller, C. B., & Fegley, S. R. (2021). Natural intertidal oyster reef growth across two landscape settings and tidal ranges. *Estuaries and Coasts*, **44**(8), 2118-2131. https//doi.org/10.1007/s12237-021-00925-2

Bracchetti, L., Capriotti, M., Fazzini, M., Cocci, P., & Palermo, F. A. (2024). Mass mortality event of mediterranean mussels (*Mytilus galloprovincialis*) in the middle Adriatic: Potential implications of the climate crisis for marine ecosystems. *Diversity*, **16**(3). https//doi.org/10.3390/d16030130

Brenner, M., Broeg, K., Wilhelm, C., Buchholz, C., & Koehler, A. (2012). Effect of air exposure on lysosomal tissues of *Mytilus edulis* L. from natural intertidal wild beds and submerged culture ropes. *Comparative Biochemistry and Physiology - A Molecular and Integrative Physiology*, **161**(3), 327-336. https//doi.org/10.1016/j.cbpa.2011.12.001

Calabrese, C., Arduini, D., Portacci, G., Quarta, E., Giangrande, A., Acquaviva, M. I., Biandolino, F., Giandomenico, S., Prato, E., & Stabili, L. (2025). Farming strategy under climate change: Growth performances and quality of *Mytilus galloprovincialis* in an Integrated Multi-Trophic Aquaculture system (North-West Mediterranean Sea). *Marine Pollution Bulletin*, **220**. https//doi.org/10.1016/j.marpolbul.2025.118377

Calabrese, C., Giangrande, A., Quarta, E., Arduini, D., Acquaviva, M. I., Biandolino, F., Giandomenico, S., Pitarra, G., Prato, E., & Stabili, L. (2026). The Mediterranean introduced pearl oyster Pinctada radiata: can an invasion be changed into a gain in a climate change scenario? *Marine Pollution* Bulletin, **222**. https//doi.org/10.1016/j.marpolbul.2025.118736

Calderwood, J., O'Connor, N. E., & Roberts, D. (2015). The effects of transportation stress and barnacle fouling on predation rates of starfish (*Asterias rubens*) on mussels (*Mytilus edulis*). *Aquaculture*, **444**, 108-113. https//doi.org/10.1016/j.aquaculture.2015.02.038

Calderwood, J., O'Connor, N. E., & Roberts, D. (2016). Breaking and entering: Examining the role of stress and aerial exposure in predator-prey relationships between the common shore crab (*Carcinus maenas*) and cultivated blue mussels (*Mytilus edulis*). *Aquaculture*, **452**, 217-223. https//doi.org/10.1016/j.aquaculture.2015.11.009

Capriotti, M., Bracchetti, L., Cocci, P., Mazzocchi, V., Fazzini, M., Corvaro, F., & Palermo, F. A. (2025). In situ investigation of ecological and molecular stress mechanisms triggered by marine heat waves in Adriatic populations of the Mediterranean mussel. *Diversity*, **17**(6). https//doi.org/10.3390/d17060385

Caputi, N., Kangas, M. I., Chandrapavan, A., Hart, A., Feng, M., Marin, M., & de Lestang, S. (2019). Factors affecting the recovery of invertebrate stocks from the 2011 Western Australian extreme marine heatwave. *Frontiers in Marine Science*, **6**. https//doi.org/10.3389/fmars.2019.00484

Carey, J. (2022). The drivers and the implications of marine heatwaves. *Proceedings of the National Academy of Sciences of the United States of America*, **119**(26). https//doi.org/10.1073/pnas.2209393119

Carneiro, A. P., Soares, C. H. L., & Pagliosa, P. R. (2021). Does the environmental condition affect the tolerance of the bivalve *Anomalocardia flexuosa* to different intensities and durations of marine heatwaves? *Marine Pollution Bulletin*, 168. https//doi.org/10.1016/j.marpolbul.2021.112410

Casas, S. M., & La Peyre, J. F. (2020). Heat shock protein 70 levels and post-harvest survival of eastern oysters following sublethal heat shock in the laboratory or conditioning in the field. Cell Stress and Chaperones, **25**(2), 369-378. https//doi.org/10.1007/s12192-019-01056-1

Castro-Olivares, A., Des, M., deCastro, M., Thomas, B., & Gómez-Gesteira, M. (2025). Assessing the vulnerability of commercial bivalves to intensifying atmospheric heatwaves in coastal ecosystems. *Marine Environmental Research*, **209**. https//doi.org/10.1016/j.marenvres.2025.107210

Chapuis, M., McDevitt-Irwin, J. M., Palmisciano, M., Elahi, R., Detrait, A., Gorum, O., Vogel, C., Chui, E., Haupt, A., Micheli, F. (2026). Recent changes in mussel beds following mass mortality of a keystone marine predator. *Marine Ecology Progress Series*, **776**. https//doi.org/10.3354/meps15006

Chen, C., Gao, J., Zhang, C., Qin, X., Cao, W., Zheng, H., & Lin, H. (2023). Cloning and mRNA expression of selenium-dependent glutathione peroxidase from the oyster *Magallana hongkongensis* in response to cadmium exposure and aerial exposure at different temperatures. *Fisheries Science*, **89**(5), 633-646. https//doi.org/10.1007/s12562-023-01704-7

Chen, H., Wang, L., Wang, L., Zhang, H., Wang, H., & Song, L. (2023). Synergistic modulation of neuroendocrine-inflammation pathway by microRNAs facilitates intertidal adaptation of molluscs. *Fish and Shellfish Immunology*, **142**. https//doi.org/10.1016/j.fsi.2023.109165

Chen, H., Xin, L., Song, X., Wang, L., Wang, W., Liu, Z., Zhang, H., Wang, L., Zhou, Z., Qui, L., & Song, L. (2017). A norepinephrine-responsive miRNA directly promotes CgHSP90AA1 expression in oyster haemocytes during desiccation. *Fish and Shellfish Immunology*, **64**, 297-307. https//doi.org/10.1016/j.fsi.2017.03.020

Chen, H., Xin, L., Wang, L., Zhang, H., Liu, R., Wang, H., Qiao, X., Wang, L., &Song, L. (2021). Ca2+/Calmodulin-NOS/NO-TNFs pathway hallmarks the inflammation response of oyster during aerial exposure. *Frontiers in Marine Science*, **7**. https//doi.org/10.3389/fmars.2020.603825

Chen, S., Zhang, C., Xiong, Y., Tian, X., Liu, C., Jeevithan, E., & Wu, W. (2015). A GC-MS-based metabolomics investigation on scallop (*Chlamys farreri*) during semi-anhydrous living-preservation. *Innovative Food Science and Emerging Technologies*, **31**, 185-195. https//doi.org/10.1016/j.ifset.2015.07.003

Cheng, M. C. F., Ericson, J. A., Ragg, N. L. C., Dunphy, B. J., & Zamora, L. N. (2025). Mussels, *Perna canaliculus*, as biosensors for climate change: concurrent monitoring of heart rate, oxygen consumption and gaping behaviour under heat stress. *New Zealand Journal of Marine and Freshwater Research*, **59**(5), 1621-1639. https//doi.org/10.1080/00288330.2025.2461757

Ciuffardi, T., Becagli, S., Raiteri, G., Bordone, A., Gabrielli, E., Magaldi, M. G., Peirano, A., Piccolin, F., Gjanci, P., Petriolo, C., Spaccini, D., Abbate, M., Rossetti, I., Varrella, P., & Lombardi, C. (2025). Physical and biogeochemical variability in the Gulf of La Spezia (Eastern Ligurian Sea, Italy): insights from a new high-resolution coastal observatory. *Journal of Marine Systems*, **252**. https//doi.org/10.1016/j.jmarsys.2025.104156

Clarke, B. C., & Griffiths, C. L. (1990). Ecological energetics of mussels *Choromytilus meridionalis* under simulated intertidal rock pool conditions. *Journal of Experimental Marine Biology and Ecology*, **137**(1), 63-77. https//doi.org/10.1016/0022-0981(90)90060-P

Clements, J. C., Harrison, S., Roussel, M., Hunt, J., Power, B. L., & Sonier, R. (2025). Fishing during extreme heatwaves alters ecological interactions and increases indirect fishing mortality in a ubiquitous nearshore system. *Communications Biology*, **8**(1). https//doi.org/10.1038/s42003-025-08158-w

Coleman, N. (1973). The oxygen consumption of *Mytilus edulis* in air. *Comparative Biochemistry and Physiology* -- Part A: Physiology, **45**(2), 393-402. https//doi.org/10.1016/0300-9629(73)90445-3

Coleman, N., & Trueman, E. R. (1971). The effect of aerial exposure on the activity of the mussels *Mytilus edulis* L. and *Modiolus modiolus* (L.). *Journal of Experimental Marine Biology and Ecology*, **7**(3), 295-304. https//doi.org/10.1016/0022-0981(71)90011-6

Connor, K., & Gracey, A. Y. (2020). Cycles of heat and aerial-exposure induce changes in the transcriptome related to cell regulation and metabolism in *Mytilus californianus*. *Marine* Biology, **167**(9). https//doi.org/10.1007/s00227-020-03750-6

Cook, K. M., Dunn, M. R., Behrens, E., Pinkerton, M. H., Law, C. S., & Cummings, V. J. (2025). The impacts of marine heatwaves on ecosystems and fisheries in Aotearoa New Zealand. *New Zealand Journal of Marine and Freshwater Research*, **59**(5), 1530-1560. https//doi.org/10.1080/00288330.2024.2436661

Copedo, J. S., Webb, S. C., Ragg, N. L. C., Ericson, J. A., Venter, L., Schmidt, A. J., Delorme, N. J., & Alfaro, A. C. (2023). Histopathological changes in the greenshell mussel, *Perna canaliculus*, in response to chronic thermal stress. *Journal of Thermal Biology*, **117**. https//doi.org/10.1016/j.jtherbio.2023.103699

Corliss, K., von Biela, V., Coletti, H., Bodkin, J., Esler, D., & Iken, K. (2024). Relative importance of macroalgae and phytoplankton to nearshore consumers and growth across climatic conditions in the Northern Gulf of Alaska. *Estuaries and Coasts*, **47**(6), 1579-1597. https//doi.org/10.1007/s12237-024-01371-6

Correia-Martins, A., Tremblay, R., Bec, B., Roques, C., Atteia, A., Gobet, A., Richare, M., Hamaguchi, M., Miyajima, T., Hori, M., Miron, G., Pouvreau, S., Lagarde, F. (2022). Failure of bivalve foundation species recruitment related to trophic changes during an extreme heatwave event. *Marine Ecology Progress Series*, **691**, 69-82. https//doi.org/10.3354/meps14060

Cowan, M. W., Pearce, C. M., Green, T. J., Finston, T., Meyer, G. R., McAmmond, B., Van Hamme, J. D., Bottos, E. M., Marshall, R., Evans, W., Sutherland, T. F., de la Bastide, P. Y. (2024). Abundance of Vibrio aestuarianus, water temperature, and stocking density are associated with summer mortality of Pacific oysters in suspended culture. *Aquaculture International*, **32**(4), 5045-5066. https//doi.org/10.1007/s10499-024-01415-5

Cruz, B., Cunha, M., Leite, C., Bordalo, D., Vieira, H. C., Soares, M. V. M., Pereira, M. E., Freitas, R. (2026). Marine heatwaves modulate the toxicity of critical metals relevant to battery technologies: Understanding the results through integrated biological response index and predictive modelling. *Journal of Hazardous Materials*, **507**. https//doi.org/10.1016/j.jhazmat.2026.141679

Cubillos, V. M., Salas-Yanquin, L. P., Mardones-Toledo, D. A., Ramírez-Kuschel, E. F., Paredes-Molina, F. J., Büchner-Miranda, J. A., & Chaparro, O. R. (2024). Location also matters: The oxidative response of the intertidal purple mussel *Perumytilus purpuratus* during tidal cycle. *Marine Environmental Research*, **199**. https//doi.org/10.1016/j.marenvres.2024.106562

Curelovich, J., Lovrich, G. A., & Calcagno, J. A. (2016). The role of the predator *Trophon geversianus* in an intertidal population of *Mytilus chilensis* in a rocky shore of the Beagle Channel, Tierra del Fuego, Argentina. *Marine Biology Research*, **12**(10), 1053-1063. https//doi.org/10.1080/17451000.2016.1228976

Curley, E. A. M., Thomas, R., Adams, C. E., & Stephen, A. (2021). Behavioural and metabolic responses of Unionida mussels to stress. *Aquatic Conservation: Marine and Freshwater Ecosystems*, **31**(11), 3184-3200. https//doi.org/10.1002/aqc.3689

Davenport, J., & Wong, T. M. (1992). Effects of temperature and aerial exposure on three tropical oyster species, *Crassostrea belcheri*, *Crassostrea iradelei* and *Saccostrea cucullata*. *Journal of Thermal Biology*, **17**(3), 135-139. https//doi.org/10.1016/0306-4565(92)90023-9

De Almeida, E. A., & Bainy, A. C. D. (2006). Effects of aerial exposure on antioxidant defenses in the brown mussel *Perna perna*. Brazilian Archives of Biology and Technology, **49**(2), 225-229. https//doi.org/10.1590/s1516-89132006000300007

de Andrade, J. T. M., Cordeiro, N. I. S., Montresor, L. C., da Luz, D. M. R., Viana, E. M. F., Martinez, C. B., & Vidigal, T. H. D. A. (2021). Tolerance of *Limnoperna fortunei* (Dunker, 1857) (Bivalvia: Mytilidae) to aerial exposure at different temperatures. *Hydrobiologia*, **848**(12), 2993-3001. https//doi.org/10.1007/s10750-020-04191-4

de Carvalho, I. S. C., Cantanhêde, S. M., Hamoy, M., da Cruz Freitas Júnior, J. R., & Amado, L. L. (2022). Cardiac responses in *Crassostrea gasar*: An experimental approach of how the tidal cycle influences the heart function of the mangrove oyster. *Comparative Biochemistry and Physiology -Part A : Molecular and Integrative Physiology*, **271**. https//doi.org/10.1016/j.cbpa.2022.111264

De Marchi, L., Vieira, L. R., Intorre, L., Meucci, V., Battaglia, F., Pretti, C., Soares, A. M. V. M., Freitas, R. (2022). Will extreme weather events influence the toxic impacts of caffeine in coastal systems? Comparison between two widely used bioindicator species. *Chemosphere*, **297**. https//doi.org/10.1016/j.chemosphere.2022.134069

De Marco, A., Baldassarro, V. A., Quadalti, C., Burato, V., Calzà, L., Giardino, L., Montroni, D., Falini, G., Graziani, G., Greggio, N., Dondi, F., Ferrari, M. G., Gatta, P. P., Parma, L, Bonaldo, A. (2025). Thermal tipping points in mediterranean mussel adhesion: molecular mechanisms and mechanical consequences of heatwave on byssus production in *Mytilus galloprovincialis*. *Scientific Reports*, **15**(1). https//doi.org/10.1038/s41598-025-08708-6

de Zwaan, A., de Bont, A. M. T., & Hemelraard, J. (1983). The role of phosphoenolpyruvate carboxykinase in the anaerobic metabolism of the sea mussel *Mytilus edulis* L. *Journal of Comparative Physiology B*, **153**(2), 267-274. https//doi.org/10.1007/BF00689630

de Zwaan, A., de Bont, A. M. T., Zurburg, W., Bayne, B. L., & Livingstone, D. R. (1983). On the role of strombine formation in the energy metabolism of adductor muscle of a sessile bivalve. *Journal of Comparative Physiology B*, **149**(4), 557-563. https//doi.org/10.1007/BF00690015

de Zwaan, A., & Eertman, R. H. M. (1996). Anoxic or aerial survival of bivalves and other euryoxic invertebrates as a useful response to environmental stress - A comprehensive review. *Comparative Biochemistry and Physiology Part C: Pharmacology, Toxicology and Endocrinology*, **113**(2), 299-312. https://doi.org/10.1016/0742-8413(95)02101-9

Delorme, N. J., King, N., Cervantes-Loreto, A., South, P. M., Baettig, C. G., Zamora, L. N., Knight, B. R., Ericson, J. A., Smith, K. F., & Ragg, N. L. C. (2024). Genetics and ontogeny are key factors influencing thermal resilience in a culturally and economically important bivalve. *Scientific Reports*, **14**(1). https//doi.org/10.1038/s41598-024-70034-0

Dereuder, E. T. R., Otto, S., Rehder, G., & Sokolova, I. M. (2025). Diel-cycling hypoxia and hypercapnia interact with the physiological and redox response of the bivalve *Mytilus edulis* to heat-wave. *Marine Environmental Research*, **209**. https//doi.org/10.1016/j.marenvres.2025.107241

Dias, M., Özkan, B., Ramos, J., Marques, A., Rosa, R., Costa, P. R., & Maulvault, A. L. (2025). Hot and toxic: Accumulation dynamics and ecotoxicological responses of mussel *Mytilus galloprovincialis* exposed to marine biotoxins during a marine heatwave. *Marine Pollution Bulletin*, **213**. https//doi.org/10.1016/j.marpolbul.2025.117629

Dievart, A. M., McQuaid, C. D., Zardi, G. I., Nicastro, K. R., & Froneman, P. W. (2023). Euendolithic infestation of mussel shells indirectly improves the thermal buffering offered by mussel beds to associated molluscs, but one size does not fit all. *Diversity*, **15**(2). https//doi.org/10.3390/d15020239

Ducker, J., Joyce, P. W. S., & Falkenberg, L. J. (2023). Mussels show capacity for persistence under, and recovery from, marine heatwaves. *Marine Biology*, **170**(10). https//doi.org/10.1007/s00227-023-04274-5

Eertman, R. H. M., Groenink, C. L. F. M. G., Sandee, B., Hummel, H., & Smaal, A. C. (1995). Response of the blue mussel *Mytilus edulis* L. following exposure to PAHs or contaminated sediment. *Marine Environmental Research*, **39**(1), 169-173. https//doi.org/10.1016/0141-1136(94)00022-H

Eertman, R. H. M., Wagenvoort, A. J., Hummel, H., & Smaal, A. C. (1993). "Survival in air" of the blue mussel *Mytilus edulis* L. as a sensitive response to pollution-induced environmental stress. *Journal of Experimental Marine Biology and Ecology*, **170**(2), 179-195. https//doi.org/10.1016/0022-0981(93)90151-D

Eertman, R. H. M., Zurburg, W., Schipper, C. A., Sandee, B., & Smaal, A. C. (1996). Effects of PCB 126 and cadmium on the anaerobic metabolism of the mussel *Mytilus edulis* L. *Comparative Biochemistry and Physiology Part C: Pharmacology, Toxicology and Endocrinology*, **113**(2), 267-272. [https://doi.org/10.1016/0742-8413(95)02101-9](https://doi.org/10.1016/0742-8413(95)02096-9)

Ericson, J. A., Laroche, O., Biessy, L., Delorme, N. J., Pochon, X., Thomson-Laing, J., Ragg, N. L. C., Smith, K. F. (2023). Differential responses of selectively bred mussels (*Perna canaliculus*) to heat stress—survival, immunology, gene expression and microbiome diversity. *Frontiers in Physiology*, **14**. https//doi.org/10.3389/fphys.2023.1265879

Ericson, J. A., Venter, L., Copedo, J. S., Nguyen, V. T., Alfaro, A. C., & Ragg, N. L. C. (2023). Chronic heat stress as a predisposing factor in summer mortality of mussels, *Perna canaliculus*. *Aquaculture*, **564**. https//doi.org/10.1016/j.aquaculture.2022.738986

Ewere, E. E., Rosic, N., Bayer, P. E., Ngangbam, A., Edwards, D., Kelaher, B. P., Mamo, L. T., Benkendorff, K. (2021). Marine heatwaves have minimal influence on the quality of adult Sydney rock oyster flesh. *Science of the Total Environment*, **795**. https//doi.org/10.1016/j.scitotenv.2021.148846

Fernández-García, F., Mieiro, C., Pacheco, M., Asturiano, J. F., & Morini, M. (2026). Characterization and environmental stress-induced expression profiling of transient receptor potential vanilloid (TRPV) channels in the Pacific oyster (*Magallana gigas*) following short-heatwave and silver exposure. *Comparative Biochemistry and Physiology Part - C: Toxicology and Pharmacology*, **304**. https//doi.org/10.1016/j.cbpc.2026.110477

Fields, P. A., Eurich, C., Gao, W. L., & Cela, B. (2014). Changes in protein expression in the salt marsh mussel *Geukensia demissa*: Evidence for a shift from anaerobic to aerobic metabolism during prolonged aerial exposure. *Journal of Experimental Biology*, **217**(9), 1601-1612. https//doi.org/10.1242/jeb.101758

Filippini, G., Bugnot, A. B., O'Connor, W., Gribben, P. E., Varkey, D. R., Paulsen, I. T., & Dafforn, K. A. (2026). Marine heatwaves alter shell microbiomes and denitrification capacity: An oyster family-specific response. *Marine Pollution Bulletin*, **227**. https//doi.org/10.1016/j.marpolbul.2026.119503

Filippini, G., Ross, P. M., O'Connor, W. A., Roberts, S., Thiyagarajan, V., Luger, C., & Parker, L. M. (2026). Transgenerational plasticity responses of larvae of Sydney rock oysters (*Saccostrea glomerata*) to ocean warming. *Marine Pollution Bulletin*, **222**. https//doi.org/10.1016/j.marpolbul.2025.118896

Fouet, M. P. A., Baudot, L., Romero-Ramirez, A., Massé, C., Blanchet, H., Bernard, G., & Maire, O. (2026). Impact of combined atmospheric and marine heatwaves on the filtration activity of the invasive Asian date mussel, *Arcuatula senhousia*. *Estuarine, Coastal and Shelf Science*, **332**. https//doi.org/10.1016/j.ecss.2026.109767

Fouet, M. P. A., Massé, C., Bonnissant, L., Blanchet, H., Maire, O., & Bernard, G. (2025). Combined atmospheric and marine heatwaves exacerbate the impacts of a non-indigenous species, the Asian date mussel *Arcuatula senhousia*, on benthic ecosystem functioning. Marine Environmental Research, **212**. https//doi.org/10.1016/j.marenvres.2025.107560

Foulk, A., Gouhier, T., Choi, F., Torossian, J. L., Matzelle, A., Sittenfeld, D., & Helmuth, B. (2024). Physiologically informed organismal climatologies reveal unexpected spatiotemporal trends in temperature. *Conservation Physiology*, **12**(1). https//doi.org/https//doi.org/10.1093/conphys/coae025

Funesto, E. G. M., Lewis, A. M., Turner, A. D., Cameron, T. C., & Steinke, M. (2023). Immediate and delayed effects of a heatwave and *Prorocentrum lima* ((Ehrenberg) Stein 1878) bloom on the toxin accumulation, physiology, and survival of the oyster *Magallana gigas* (Thunberg, 1793). *Science of the Total Environment*, **892**. https//doi.org/10.1016/j.scitotenv.2023.164485

Galil, B. S., Mienis, H. K., Mendelson, M., Gayer, K., & Goren, M. (2022). Here today, gone tomorrow – the Levantine population of the Brown mussel *Perna perna* obliterated by unprecedented heatwave. *Aquatic Invasions*, **17**(2), 174-185. https//doi.org/10.3391/AI.2022.17.2.03

García-Souto, D., Martínez-Mariño, V., Morán, P., Olabarria, C., & Vázquez, E. (2024). Hiding from heat: The transcriptomic response of two clam species is modulated by behaviour and habitat. *Journal of Thermal Biology*, **119**. https//doi.org/10.1016/j.jtherbio.2023.103776

Geng, J., Yan, H., Liu, C., Zhao, N., Liu, S., Han, T., Dodson, J., Wen, H. (2025). Adaptation and challenges for giant clam species under marine heatwaves. *Global and Planetary Change*, **248**. https//doi.org/10.1016/j.gloplacha.2025.104746

George, M. N., Cattau, O., Middleton, M. A., Lawson, D., Vadopalas, B., Gavery, M., & Roberts, S. B. (2023). Triploid Pacific oysters exhibit stress response dysregulation and elevated mortality following heatwaves. *Global Change Biology*, **29**(24), 6969-6987. https//doi.org/10.1111/gcb.16880

Gerraty, F. D., Cox-Ammann, K. N., Douglas, M. A., George, M., Lohse, D. P., Miner, C. M., & Raimondi, P. T. (2026). Marine heatwave and keystone predator loss drive broad-scale decline and hinder recovery of a rocky intertidal kelp. *Ecological Applications*, **36**(2). https//doi.org/10.1002/eap.70215

Gillmor, R. B. (1982). Assessment of intertidal growth and capacity adaptations in suspension-feeding bivalves. *Marine Biology*, **68**(3), 277-286. https//doi.org/10.1007/BF00409594

Gilson, A. R., Coughlan, N. E., Dick, J. T. A., & Kregting, L. (2021). Marine heat waves differentially affect functioning of native (*Ostrea edulis*) and invasive (*Crassostrea [Magallana] gigas*) oysters in tidal pools. *Marine Environmental Research*, **172**. https//doi.org/10.1016/j.marenvres.2021.105497

Götze, S., Reddin, C. J., Ketelsen, I., Busack, M., Lannig, G., Bock, C., & Pörtner, H. O. (2025). Cardiac performance mirrors the passive thermal tolerance range in the oyster *Ostrea edulis*. *Journal of Experimental Biology*, 228(2). https//doi.org/10.1242/jeb.249750

Greenhough, H., Smith, K. F., Vignier, J., Miller, M. R., Passfield, E., Kenny, N. J., & Rolton, A. (2025). Impacts of marine heatwave stress and harmful algal (*Alexandrium spp*.) exposure on subadult green-lipped mussels (*Perna canaliculus*). *Ecotoxicology and Environmental Safety*, **304**. https//doi.org/10.1016/j.ecoenv.2025.119112

Greenhough, H., Vignier, J., Smith, K. F., Brown, C. M., Kenny, N. J., & Rolton, A. (2025). Multi-stressor dynamics: Effects of marine heatwave stress and harmful algal blooms on juvenile mussel (*Perna canaliculus*) survival and physiology. *Science of the Total Environment*, **964**. https//doi.org/10.1016/j.scitotenv.2025.178590

Griffiths, R. J. (1981). Aerial exposure and energy balance in littoral and sublittoral *Choromytilus meridionalis* (Kr.) (Bivalvia). *Journal of Experimental Marine Biology and Ecology*, **52**(2), 231-241. https//doi.org/10.1016/0022-0981(81)90039-3

Grimmelpont, M., Payton, L., Lefrançois, C., & Tran, D. (2024). Molecular and behavioural responses of the mussel *Mytilus edulis* exposed to a marine heatwave. *Marine Environmental Research*, **196**. https//doi.org/10.1016/j.marenvres.2024.106418

Guareschi, S., & Wood, P. J. (2020). Exploring the desiccation tolerance of the invasive bivalve *Corbicula fluminea* (Müller 1774) at different temperatures. *Biological Invasions*, **22**(9), 2813-2824. https//doi.org/10.1007/s10530-020-02291-9

Guo, S., Li, J., Yang, X., Qin, Y., Zhao, Y., Wei, J., Ma, H., Yu, Z., Zhao, L., Zhang, Y. (2024). Resistance of an intertidal oyster (*Saccostrea mordax*) to marine heatwaves and the implication for reef building. *Science of the Total Environment*, **928**. https//doi.org/10.1016/j.scitotenv.2024.172474

Hanekom, N. (2011). Trophic structure and biomass distribution of macrobenthos on sheltered and semi-exposed rocky shores of Tsitsikamma Marine Protected Area. *African Zoology*, **46**(2), 224-238. https//doi.org/10.3377/004.046.0203

He, G., Liu, X., Xu, Y., Liang, J., Deng, Y., Zhang, Y., & Zhao, L. (2021). Repeated exposure to simulated marine heatwaves enhances the thermal tolerance in pearl oysters. *Aquatic Toxicology*, **239**. https//doi.org/10.1016/j.aquatox.2021.105959

He, G., Liu, X., Xu, Y., Liu, Y., Masanja, F., Deng, Y., & Zhao, L. (2024). Metabolic dysfunctions in pearl oysters following recurrent marine heatwaves. *Marine Environmental Research*, 200. https//doi.org/10.1016/j.marenvres.2024.106641

He, G., Peng, Y., Liu, X., Liu, Y., Liang, J., Xu, X., Yang, K., Masanja, F., Xu, Y., Deng, Y., Zhao, L. (2022). Post-responses of intertidal bivalves to recurrent heatwaves. *Marine Pollution Bulletin*, **184**. https//doi.org/10.1016/j.marpolbul.2022.114223

He, G., Xiong, X., Peng, Y., Yang, C., Xu, Y., Liu, X., Liang, J., Masanja, F., Yang, K., Xu, X., Zheng, Z., Deng, Y., Leung., J. Y. S., Zhao, L. (2023). Transcriptomic responses reveal impaired physiological performance of the pearl oyster following repeated exposure to marine heatwaves. *Science of the Total Environment*, **854**. https//doi.org/10.1016/j.scitotenv.2022.158726

He, G., Zou, J., Liu, X., Liang, F., Liang, J., Yang, K., Masanja, F., Xu, Y, Zheng, Z., Deng, Y., Zhao, L. (2022). Assessing the impact of atmospheric heatwaves on intertidal clams. *Science of the Total Environment*, **841**. https//doi.org/10.1016/j.scitotenv.2022.156744

He, Y., Xu, Y., Liang, H., Liu, Y., Deng, Y., Masanja, F., & Zhao, L. (2026). Assessing impacts of extreme climate and weather events on endangered pearl oysters *Pinctada maxima*. *Marine Environmental Research*, **215**. https//doi.org/10.1016/j.marenvres.2025.107821

Helmuth, B. (1999). Thermal biology of rocky intertidal mussels: Quantifying body temperatures using climatological data. *Ecology*, **80**(1), 15-34. https//doi.org/10.1890/0012-9658(1999)080[0015:TBORIM]2.0.CO;2

Helmuth, B., Broitman, B. R., Blanchette, C. A., Gilman, S., Halpin, P., Harley, C. D. G., O’Donnell, M. J., Hofmann, G. E., Menge, B., Strickland, D. (2006). Mosaic patterns of thermal stress in the rocky intertidal zone: Implications for climate change. Ecological Monographs, **76**(4), 461-479. https//doi.org/10.1890/0012-9615(2006)076[0461:MPOTSI]2.0.CO;2

Helmuth, B. S. T. (1998). Intertidal mussel microclimate: Predicting the body temperature of a sessile invertebrate. *Ecological Monographs*, **68**(1), 51-74. https//doi.org/10.1890/0012-9615(1998)068[0051:IMMPTB]2.0.CO;2

Hiong, K. C., Peh, W. Y. X., Loong, A. M., Wong, W. P., Chew, S. F., & Ip, Y. K. (2004). Exposure to air, but not seawater, increases the glutamine content and the glutamine synthetase activity in the marsh clam *Polymesoda expansa*. *Journal of Experimental Biology*, **207**(26), 4605-4614. https//doi.org/https//doi.org/10.1242/jeb.01334

Holwerda, D. A., Veenhof, P. R., Van Heugten, H. A. A., & Zandee, D. I. (1983). Modification of mussel pyruvate kinase during anaerobiosis and after temperature acclimation. *Molecular Physiology*, **3**(3), 225-234.

Hong, H.-K., Kim, C. W., Kim, J.-H., Kajino, N., & Choi, K.-S. (2021). Effect of extreme heatwaves on the mortality and cellular immune responses of purplish bifurcate mussel *Mytilisepta virgata* (Wiegmann, 1837) (=*Septifer virgatus*) in indoor mesocosm experiments. *Frontiers in Marine Science*, **8**. https//doi.org/10.3389/fmars.2021.794168

Hook, S. E., Hobday, A. J., Farr, R. J., Su, J., Court, L., Pandey, G., Rane, R. V., Kangas, M., Bermudes, M., Fontanini, A., Walsh, T. (2025). Genomic resources and transcriptomic evidence of thermal stress in the southern saucer scallop (*Ylistrum balloti*). *Comparative Biochemistry and Physiology - Part D: Genomics and Proteomics*, **56**. https//doi.org/10.1016/j.cbd.2025.101571

Howarth, N., Scanes, E., Byrne, M., & Ross, P. M. (2024). Ocean warming and Marine Heatwaves unequally impact juvenile introduced and native oysters with implications for their coexistence and future distribution. *Scientific Reports*, **14**(1). https//doi.org/10.1038/s41598-024-71534-9

Iuffrida, L., & Franzellitti, S. (2025). Transcriptional responses of Mediterranean mussels (*Mytilus galloprovincialis*) under the 2022 Marine Heatwave: a trade-off of physiological regulation between metabolism, stress response, and shell biomineralization in a mixed exposure scenario. *Environmental Pollution*, **375**. https//doi.org/10.1016/j.envpol.2025.126328

Iuffrida, L., Spezzano, R., Trapella, G., Cinti, N., Parma, L., De Marco, A., Palladino, G., Bonaldo, A., Candela, M., Franzellitti, S. (2024). Physiological plasticity and life history traits affect *Chamelea gallina* acclimatory responses during a marine heatwave. *Environmental Research*, **263**. https//doi.org/10.1016/j.envres.2024.120287

James Bond, J. A., Escobar, P. I., Hood, N. Z., Hong, H. C., Rankins, D. R., Chan, G., Betancourt, J. V., French, K. B., Procter, N. D., Wehrle. B. A., Connor, K. M. (2026). Mussels enhance digestive enzyme activity in preparation for stressful fluctuating environments. *Journal of Comparative Physiology B: Biochemical, Systemic, and Environmental Physiology*, **196**(1), 1-16. https//doi.org/10.1007/s00360-025-01637-w

Jiang, K., Nie, H., & Yan, X. (2020). Molecular cloning and expression analysis of mc5r like genes (mc5rl) in *Ruditapes philippinarum* (Manila clam) after aerial exposure and low-temperature stress. *Molecular Biology Reports*, **47**(11), 8891-8901. https//doi.org/10.1007/s11033-020-05941-2

Jiang, X., Zhang, X., Guan, J., He, P., Wei, P., Zhang, L., Peng, J., Zhao, L. (2025). Physiological energetics of selectively bred oysters (*Crassostrea hongkongensis*) under marine heatwaves. *Marine Environmental Research*, **204**. https//doi.org/10.1016/j.marenvres.2024.106871

Jokumsen, A., & Fyhn, H. J. (1982). The influence of aerial exposure upon respiratory and osmotic properties of haemolymph from two intertidal mussels, *Mytilus edulis* L. and *Modiolus modiolus* L. *Journal of Experimental Marine Biology and Ecology*, **61**(2), 189-203. https//doi.org/10.1016/0022-0981(82)90008-9

Jolma, E. R., van Leeuwen, S. M., & Thieltges, D. W. (2026). Thermal buffering by intertidal oyster reefs on mudflats in the European Wadden Sea. *Marine Environmental Research*, **215**. https//doi.org/10.1016/j.marenvres.2026.107834

Jones, S. J., Lima, F. P., & Wethey, D. S. (2010). Rising environmental temperatures and biogeography: Poleward range contraction of the blue mussel, *Mytilus edulis* L., in the western Atlantic. *Journal of Biogeography*, **37**(12), 2243-2259. https//doi.org/10.1111/j.1365-2699.2010.02386.x

Kajtar, J. B., Holbrook, N. J., Lyth, A., Hobday, A. J., Mundy, C. N., & Ugalde, S. C. (2024). A stakeholder-guided marine heatwave hazard index for fisheries and aquaculture. *Climatic Change*, **177**(2). https//doi.org/10.1007/s10584-024-03684-8

Kangas, M., Chandrapavan, A., Wilkin, S., & Caputi, N. (2022). Recovery trajectories and management responses for three scallop stocks over ten years following an extreme marine heatwave in Western Australia. *Frontiers in Climate,* **4**. https//doi.org/10.3389/fclim.2022.1043889

Karatayev, A. Y., Burlakova, L. E., & Padilla, D. K. (1998). Physical factors that limit the distribution and abundance of *Dreissena polymorpha* (Pall.). *Journal of Shellfish Research*, **17**(4), 1219-1235.

King, N. G., Leathers, T., Smith, K. E., & Smale, D. A. (2025). The influence of pre-exposure to marine heatwaves on the critical thermal maxima (CTmax) of marine foundation species. *Functional Ecology*, **39**(8), 1869-1878. https//doi.org/10.1111/1365-2435.14622

Kingsley-Smith, P. R., & Luckenbach, M. W. (2008). Post-settlement survival and growth of the suminoe oyster, *Crassostrea ariakensis*, exposed to simulated emersion regimes. *Journal of Shellfish Research*, **27**(3), 609-618. https//doi.org/10.2983/0730-8000(2008)27[609:PSAGOT]2.0.CO;2

Kozal, L. C., Hofmann, G. E., Hawes, N. A., & Ragg, N. L. C. (2024). Paternal heat exposure affects progeny larval development in green-lipped mussels *Perna canaliculus*. *Aquaculture Environment Interactions*, **16**, 43-57. https//doi.org/10.3354/AEI00472

Lam-Gordillo, O., Douglas, E. J., Hailes, S. F., Cummings, V., & Lohrer, A. M. (2025). Effects of in situ experimental warming on metabolic expression in a soft sediment bivalve. *Scientific Reports*, **15**(1). https//doi.org/10.1038/s41598-025-86310-6

Lattos, A., Papadopoulos, D. K., Feidantsis, K., Giantsis, I. A., Georgoulis, I., Karagiannis, D., & Michaelidis, B. (2023). Antioxidant defense of *Mytilus galloprovincialis* mussels induced by marine heatwaves in correlation with marteilia pathogen presence. *Fishes*, **8**(8). https//doi.org/10.3390/fishes8080408

Lattos, A., Papadopoulos, D. K., Feidantsis, K., Karagiannis, D., Giantsis, I. A., & Michaelidis, B. (2022). Are marine heatwaves responsible for mortalities of farmed *Mytilus galloprovincialis*? A pathophysiological analysis of marteilia Infected mussels from Thermaikos Gulf, Greece. animals, **12**(20). https//doi.org/10.3390/ani12202805

Liang, J., Masanja, F., Li, Y., Guo, Y., Deng, Y., & Zhao, L. (2024). Hard clam resilience to marine heatwaves in the face of climate change. *Frontiers in Marine* Science, **11**. https//doi.org/10.3389/fmars.2024.1382825

Littlewood, D. T. J. (1988). Subtidal versus intertidal cultivation of *Crassostrea rhizophorae*. *Aquaculture*, **72**(1), 59-71. https//doi.org/10.1016/0044-8486(88)90146-9

Liu, B., Hu, M., Li, L., Zhang, M., Li, X., Abo-Raya, M. H., & Wang, Y. (2026). Combined effects of marine heatwaves and nano-titanium dioxide (nano-TiO₂) on the physiological fitness in the mussel *Mytilus coruscus*. *Marine Pollution Bulletin*, **222**. https//doi.org/10.1016/j.marpolbul.2025.118659

Liu, D., Zhu, B., Liang, Q., Zhang, H., Dong, S., & Wang, F. (2023). High temperatures enhance the strength of multiple predator effects in a typical crab-clam system. *Marine Pollution Bulletin*, **188**. https//doi.org/10.1016/j.marpolbul.2023.114670

Liu, M., Wang, Z., Ni, H., Zhuo, W., Yuan, G., & Dong, Z. (2025). Adaptability of clams to prolonged heatwaves: Influences on gene expression, antioxidant capacity, and histology of Chinese cyclina *Cyclina sinensis*. *Aquaculture Reports*, **40**. https//doi.org/10.1016/j.aqrep.2024.102559

Liu, X., Peng, Y., Xu, Y., He, G., Liang, J., Masanja, F., Yang, K., Xu, X., Deng, Y., Zhao, L. (2023). Responses of digestive metabolism to marine heatwaves in pearl oysters. *Marine Pollution Bulletin*, **186**. https//doi.org/10.1016/j.marpolbul.2022.114395

Liu, Y., Ren, J. S., Wang, X., Chen, J., Wu, W., & Zhang, J. (2025). The impact of marine heatwaves and hypoxia on behavior and physiology of Manila clam *Ruditapes philippinarum*. *Marine Pollution* Bulletin, **219**. https//doi.org/10.1016/j.marpolbul.2025.118327

Liu, Y., Wang, Y., Liu, S., Lin, Z., & Xue, Q. (2026). Short-term heat stress adaptation in intertidal oysters (*Crassostrea sikamea*): Integrative biochemical, transcriptomic and metabolomic insights. *Journal of Thermal Biology*, **135**. https//doi.org/10.1016/j.jtherbio.2025.104355

Liu, Y., Zhang, J., Wang, X., Wu, W., Kang, Q., & Li, C. (2022). Temperature-Induced Environmental Chain Reaction in Marine Sedimentation and Its Impact on Manila Clam *Ruditapes philippinarum*. *Frontiers in Marine Science*, **9**. https//doi.org/10.3389/fmars.2022.845768

Lugue, K., Monaco, C. J., Vigouroux, E., Koua, M. S., Vidal-Dupiol, J., Mitta, G., & Le Luyer, J. (2025). Exploring thermal tolerance across time and space in a tropical bivalve, *Pinctada margaritifera*. *Journal of Experimental Biology*, **228**(5). https//doi.org/10.1242/jeb.249651

Luo, X., Masanja, F., Liu, Y., & Zhao, L. (2025). Behavioral responses of clams to recurrent marine heatwaves. *Marine Pollution Bulletin*, **210**. https//doi.org/10.1016/j.marpolbul.2024.117362

Luo, X., Zhang, X., Xu, Y., Masanja, F., Yang, K., Liu, Y., & Zhao, L. (2024). Behavioral responses of intertidal clams to compound extreme weather and climate events. *Marine Pollution* Bulletin, **200**. https//doi.org/10.1016/j.marpolbul.2024.116112

Ma, L.-X., Wang, J., Denny, M., & Dong, Y.-W. (2024). Hindcasted body temperatures reveal underestimated thermal stress faced by intertidal species. *Global Ecology and Biogeography*, **33**(11). https//doi.org/10.1111/geb.13908

Mackenzie, C. L., Raap, M. R., Kellogg, C. T. E., Walker, C. Y. V., Clemente-Carvalho, R. B. G., Green, T. J., & Pearce, C. M. (2025). A common garden comparison of the microbiome and gene expression of intertidally- and subtidally-cultured Pacific oysters (*Crassostrea gigas*) in relation to extreme warming events. *Aquaculture International*, **33**(5). https//doi.org/10.1007/s10499-025-01959-0

Mackenzie, C. L., Raap, M. R., Leduc, S., Walker, C. Y. V., Green, T. J., Kim, E., Montgomery, E. M., Gray, S. L. M., Long, A., Pearce, C. M. (2024). Development of a nature-based solution for mitigation of Pacific oyster summer mortality: use of the intertidal zone to improve resilience to environmental stressors. *Frontiers in Marine Science*, **11**. https//doi.org/10.3389/fmars.2024.1345493

Malek, J. C., & Breitburg, D. L. (2016). Effects of air-exposure gradients on spatial infection patterns of *Perkinsus marinus* in the eastern oyster *Crassostrea virginica*. *Diseases of Aquatic Organisms*, **118**(2), 139-151. https//doi.org/10.3354/dao02964

Manahan, D. T. (2023). Marine heatwaves impact mortality of triploid Pacific oysters. *Global Change Biology*, **29**(24), 6829-6831. https//doi.org/10.1111/gcb.16980

Marrocco, T., Petrucciani, A., Calcinai, B., Puce, S., Cerrano, C., & Norici, A. (2026). “The hidden Filter”: Quantifying the ecological role of *Lithophaga lithophaga* (Linnaeus, 1758) in Mediterranean benthic-pelagic processes. Marine Environmental Research, **213**. https//doi.org/10.1016/j.marenvres.2025.107641

Marsden, I. D., & Weatherhead, M. A. (1998). Effects of aerial exposure on oxygen consumption by the New Zealand mussel *Perna canaliculus* (Gmelin, 1791) from an intertidal habitat. *Journal of Experimental Marine Biology and Ecology*, **230**(1), 15-29. https//doi.org/10.1016/S0022-0981(98)00067-7

Masanja, F., Jiang, X., He, G., Xu, Y., Zang, X., He, Y., & Zhao, L. (2024). Bivalves under extreme weather events: A comparative study of five economically important species in the South China sea during marine heatwaves. *Marine Environmental Research*, **202**. https//doi.org/10.1016/j.marenvres.2024.106716

Masanja, F., Luo, X., Jiang, X., Xu, Y., Mkuye, R., Liu, Y., & Zhao, L. (2024). Elucidating responses of the intertidal clam *Ruditapes philippinarum* to compound extreme oceanic events. *Marine Pollution Bulletin*, **204**. https//doi.org/https//doi.org/10.1016/j.marpolbul.2024.116523

Masanja, F., Xu, Y., He, G., Liang, F., Liu, X., Yang, K., Mkuye, R., Liang, J., Deng, Y., Zhao, L. (2022). Exploring HSP90 as a biomarker for marine heatwaves in *Pinctada maxima*. *Frontiers in Marine Science*, **9**. https//doi.org/10.3389/fmars.2022.913920

Mathew, S., & Damodaran, R. (1997). Carotenoids and anoxic/hypoxic stress in bivalves *Sunetta scripta* and *Perna viridis*. *Indian Journal of Experimental Biology*, **35**(2), 176-179.

McAfee, D., O'Connor, W. A., & Bishop, M. J. (2017). Fast-growing oysters show reduced capacity to provide a thermal refuge to intertidal biodiversity at high temperatures. *Journal of Animal Ecology*, **86**(6), 1352-1362. https//doi.org/10.1111/1365-2656.12757

McFarland, K., Baker, S., Baker, P., Rybovich, M., & Volety, A. K. (2015). Temperature, salinity, and aerial exposure tolerance of the invasive mussel, *Perna viridis*, in estuarine habitats: Implications for spread and competition with native oysters, *Crassostrea virginica*. *Estuaries and Coasts*, **38**(5), 1619-1628. https//doi.org/10.1007/s12237-014-9903-5

Mendiolar, M., Filar, J. A., Yang, W.-H., Leahy, S., & Courtney, A. J. (2023). Capturing episodic impacts of environmental signals. *Environmental Modelling and Software*, **170**. https//doi.org/10.1016/j.envsoft.2023.105837

Menge, B. A., Robinson, J. W., Poirson, B. N., & Gravem, S. A. (2023). Quantitative biogeography: Decreasing and more variable dynamics of critical species in an iconic meta-ecosystem. *Ecological Monographs*, **93**(1). https//doi.org/10.1002/ecm.1556

Minaudo, C., Abonyi, A., Leitão, M., Lançon, A. M., Floury, M., Descy, J. P., & Moatar, F. (2021). Long-term impacts of nutrient control, climate change, and invasive clams on phytoplankton and cyanobacteria biomass in a large temperate river. *Science of the Total Environment*, **756**. https//doi.org/10.1016/j.scitotenv.2020.144074

Miner, C. M., Berry, H. D., Bohlmann, H., Dethier, M. N., Fradkin, S. C., Gaddam, R., Raymond, W. W., & Raimondi, P. T. (2025). Location and natural history are key to determining impact of the 2021 atmospheric heatwave on Pacific Northwest rocky intertidal communities. *Frontiers in Marine Science*, **12**. https//doi.org/10.3389/fmars.2025.1503019

Miner, C. M., Burnaford, J. L., Ammann, K., Becker, B. H., Fradkin, S. C., Ostermann-Kelm, S., Smith, J. R., Whitaker, S, G., Raimondi, P. T. (2021). Latitudinal variation in long-term stability of North American rocky intertidal communities. *Journal of Animal Ecology*, **90**(9), 2077-2093. https//doi.org/10.1111/1365-2656.13504

Monsinjon, J. R., McQuaid, C. D., Nicastro, K. R., Seuront, L., Oróstica, M. H., & Zardi, G. I. (2021). Weather and topography regulate the benefit of a conditionally helpful parasite. *Functional Ecology*, **35**(12), 2691-2706. https//doi.org/10.1111/1365-2435.13939

Montecinos, L. A., Cisterna, J. A., Cáceres, C. W., & Saldías, G. S. (2009). Acid-base balance during air exposure in the bivalve mollusk *Perumytilus purpuratus* (Lamarck, 1819) (Bivalvia: Mytilidae). *Revista de Biologia Marina y Oceanografia*, **44**(1), 181-187. https//doi.org/https//doi.org/

Montory, J. A., Chaparro, O. R., Salas-Yanquin, L. P., Buchner-Miranda, J. A., Pechenik, J. A., & Cubillos, V. M. (2021). Impact of intertidal distribution on the physiological performance of the filter-feeder bivalve *Perumytilus purpuratus* (Bivalvia, Mytilidae) from Southern Chile. *Malacologia*, **64**(1), 137-147. https//doi.org/10.4002/040.064.0108

Moreira, D. C., da Costa Tavares Sabino, M. A., Minari, M., Kuzniewski, F. T. B., Angelini, R., & Hermes-Lima, M. (2023). The role of solar radiation and tidal emersion on oxidative stress and glutathione synthesis in mussels exposed to air. *PeerJ*, 11. https//doi.org/10.7717/PEERJ.15345

Moreira, D. C., Sabino, M. A. C. T., Kuzniewski, F. T. B., Furtado-Filho, O. V., Carvajalino-Fernández, J. M., Angelini, R., Freire, C. A., & Hermes-Lima, M. (2021). Redox metabolism in mussels (*Brachidontes solisianus*) under the influence of tides in a rocky beach in Southern Brazil. Estuarine, *Coastal and Shelf Science*, **258**. https//doi.org/10.1016/j.ecss.2021.107424

Mouthon, J., & Daufresne, M. (2006). Effects of the 2003 heatwave and climatic warming on mollusc communities of the Saône: A large lowland river and of its two main tributaries (France). *Global Change Biology*, **12**(3), 441-449. https//doi.org/10.1111/j.1365-2486.2006.01095.x

Nardi, A., Benedetti, M., Gorbi, S., & Regoli, F. (2021). Interactive Immunomodulation in the Mediterranean mussel *Mytilus galloprovincialis* under thermal stress and cadmium exposure. *Frontiers in Marine Science*, **8**. https//doi.org/10.3389/fmars.2021.751983

Nardi, A., Mezzelani, M., Costa, S., d'Errico, G., Benedetti, M., Gorbi, S., Freitas, R., Regoli, F. (2022). Marine heatwaves hamper neuro-immune and oxidative tolerance toward carbamazepine in *Mytilus galloprovincialis*. *Environmental Pollution*, **300**. https//doi.org/10.1016/j.envpol.2022.118970

Negishi, J. N., & Kayaba, Y. (2009). Effects of handling and density on the growth of the unionoid mussel *Pronodularia japanensis*. *Journal of the North American Benthological Society*, **28**(4), 821-831. https//doi.org/10.1899/08-129.1

Nie, H., Dong, S., Li, D., Zheng, M., Jiang, L., Li, X., & Yan, X. (2018). RNA-Seq analysis of differentially expressed genes in the grand jackknife clam Solen grandis under aerial exposure. *Comparative Biochemistry and Physiology - Part D: Genomics and Proteomics*, **28**, 54-62. https//doi.org/10.1016/j.cbd.2018.06.003

Nie, H., Jiang, K., Li, N., Li, D., & Yan, X. (2020). Transcriptomic analysis of *Ruditapes philippinarum* under aerial exposure and reimmersion reveals genes involved in stress response and recovery capacity of the Manila clam. *Aquaculture*, 524. https//doi.org/10.1016/j.aquaculture.2020.735271

Nie, H., Jiang, K., Zhou, Z., Guo, B., Li, D., & Yan, X. (2020). Modulated expression and activities of *Ruditapes philippinarum* enzymes After oxidative stress Induced by aerial exposure and reimmersion. *Frontiers in Physiology*, **11**. https//doi.org/10.3389/fphys.2020.00500

Nogueira, L., Mello, D. F., Trevisan, R., Garcia, D., da Silva Acosta, D., Dafre, A. L., & de Almeida, E. A. (2017). Hypoxia effects on oxidative stress and immunocompetence biomarkers in the mussel *Perna perna* (Mytilidae, Bivalvia). Marine Environmental Research, **126**, 109-115. https//doi.org/10.1016/j.marenvres.2017.02.009

Normand, J., Le Pennec, M., & Boudry, P. (2008). Comparative histological study of gametogenesis in diploid and triploid Pacific oysters (*Crassostrea gigas*) reared in an estuarine farming site in France during the 2003 heatwave. *Aquaculture*, **282**(1), 124-129. https//doi.org/10.1016/j.aquaculture.2008.06.026

Ojeda, M., Boy, C. C., & Giménez, J. (2019). Reproductive aspects and energy allocation compromise to aerial exposure in the intertidal mussel *Brachidontes rodriguezii*. A case study at two tidal levels. *Journal of Sea Research*, **155**. https//doi.org/10.1016/j.seares.2019.101824

Olabarria, C., Gestoso, I., Lima, F. P., Vázquez, E., Comeau, L. A., Gomes, Seabra, R., F., & Babarro, J. M. F. (2016). Response of two mytilids to a heatwave: The complex interplay of physiology, behaviour and ecological interactions. *PLOS ONE*, **11**(10). https//doi.org/10.1371/journal.pone.0164330

Ory, P., Bonnet, A., Mondeguer, F., Breitwieser, M., Dubillot, E., & Graber, M. (2019). Metabolomics based on UHPLC-QToF- and APGC-QToF-MS reveals metabolic pathways reprogramming in response to tidal cycles in the sub-littoral species *Mimachlamys varia* exposed to aerial emergence. *Comparative Biochemistry and Physiology - Part D: Genomics and Proteomics*, **29**, 74-85. https//doi.org/10.1016/j.cbd.2018.11.002

Overton, K., Dempster, T., Swearer, S. E., Morris, R. L., & Barrett, L. T. (2024). Aerial exposure tolerance of juvenile flat oysters (*Ostrea angasi*) depends on shell length and air temperature. *Restoration Ecology*, **32**(1). https//doi.org/10.1111/rec.14047

Papadopoulos, D. K., Georgoulis, I., Feidantsis, K., Michaelidis, B., & Giantsis, I. A. (2025). Energy metabolism under thermal stress in *Mytilus galloprovincialis* and *Ruditapes decussatus*: Insights from gene expression and enzyme activity profiles. *Marine Environmental Research*, **210**. https//doi.org/10.1016/j.marenvres.2025.107349

Parizadeh, L., Saint-Picq, C., Barbier, P., Bringer, A., Huet, V., Dubillot, E., & Thomas, H. (2025). Groundbreaking study: Combined effect of marine heatwaves and polyethylene microplastics on Pacific oysters, *Crassostrea gigas*. *Environmental Pollution*, **364**. https//doi.org/10.1016/j.envpol.2024.125164

Patil, S. V., Singh, H., & Yusufzai, S. I. (2003). Effect of salinity and aerial exposure on survival of the venerid clam, *Paphia malbarica* (Chemnitz). Asian Journal of Microbiology, Biotechnology and Environmental Sciences, **5**(4), 427-429.

Paukstis, G. L., Tucker, J. K., Bronikowski, A. M., & Janzen, F. J. (1999). Survivorship of aerially-exposed zebra mussels (*Dreissena polymorpha*) under laboratory conditions. *Journal of Freshwater Ecology*, **14**(4), 511-517. https//doi.org/10.1080/02705060.1999.9663709

Pereira, R. R. C., Scanes, E., Parker, L. M., Byrne, M., Cole, V. J., & Ross, P. M. (2019). Restoring the flat oyster *Ostrea angasi* in the face of a changing climate. *Marine Ecology Progress Series*, **625**, 27-39. https//doi.org/10.3354/meps13047

Peruzza, L., Tucci, C. F., Frizzo, R., Riello, T., Quagliariello, A., Martino, M. E., Manuzzi, A., Rovere, G. D., Bonsembiante, F., Gelain, M. E., Smits, M., Borgheresi, F. C., Panin, M., Venier, P., Mammi, S., Hauton, C., Patarnello, T., Milan, M., Bargelloni, L. (2023). Impaired reproduction, energy reserves and dysbiosis: The overlooked consequences of heatwaves in a bivalve mollusc. *Marine Pollution Bulletin*, **193**. https//doi.org/10.1016/j.marpolbul.2023.115192

Peterson, E. A., Keur, M. C., Yeboah, M., van de Grootevheen, T., Moth, L., Kamermans, P., Murk, T., Peck, M. A., & Foekema, E. (2025). Determining physiological responses of mussels (*Mytilus edulis*) to hypoxia by combining multiple sensor techniques. *Conservation Physiology*, **13**(1). https//doi.org/10.1093/conphys/coaf023

Petes, L. E., Menge, B. A., & Harris, A. L. (2008). Intertidal mussels exhibit energetic trade-offs between reproduction and stress resistance. *Ecological Monographs*, **78**(3), 387-402. https//doi.org/10.1890/07-0605.1

Petrović, S., Semenčić, L., Ozretić, B., & Ozretić, M. (2004). Seasonal variations of physiological and cellular biomarkers and their use in the biomonitoring of north Adriatic coastal waters (Croatia). *Marine Pollution Bulletin*, **49**(9), 713-720. https//doi.org/10.1016/j.marpolbul.2004.05.004

Pham, T., Hong, H. C., Swig, B., German, D. P., & Connor, K. M. (2023). Elevated aerial temperature modulates digestive enzyme activities in *Mytilus californianus*. *Comparative Biochemistry and Physiology Part - B: Biochemistry and Molecular Biology*, **265**. https//doi.org/10.1016/j.cbpb.2022.110825

Pittura, L., Nardi, A., Cocca, M., De Falco, F., d’Errico, G., Mazzoli, C., Mongera, F., Benedetti, M., Gorbi, S., Avella, M., & Regoli, F. (2022). Cellular disturbance and thermal stress response in mussels exposed to synthetic and natural microfibers. *Frontiers in Marine Science*, **9**. https//doi.org/10.3389/fmars.2022.981365

Plumlee, J. D., Powers, S. P., Kimbro, D. L., Lehrter, J. C., Herrmann, J., & Mareska, J. (2024). Increasing duration of heatwaves poses a threat to oyster sustainability in the Gulf of Mexico. *Ecological Indicators*, **162**. https//doi.org/10.1016/j.ecolind.2024.112015

Rato, A., Joaquim, S., Matias, A. M., Roque, C., Marques, A., & Matias, D. (2022). The impact of climate change on bivalve farming: Combined effect of temperature and salinity on survival and feeding behavior of clams *Ruditapes decussatus*. *Frontiers in Marine Science*, **9**. https//doi.org/10.3389/fmars.2022.932310

Raymond, W. W., Barber, J. S., Dethier, M. N., Hayford, H. A., Harley, C. D. G., King, T. L., Paul, B., Speck, C. A., Tobin, E.D., Raymond, A. E. T., & McDonald, P. S. (2022). Assessment of the impacts of an unprecedented heatwave on intertidal shellfish of the Salish Sea. *Ecology*, **103**(10). https//doi.org/10.1002/ecy.3798

Raymond, W. W., Tobin, E. D., Barber, J. S., Hayford, H. A., Raymond, A. E. T., Speck, C. A., Rogers, D., & Brown, R. (2024). Short-term effects of an unprecedented heatwave on intertidal bivalve populations: fisheries management surveys provide an incomplete picture. *Frontiers in Marine Science*, **11**. https//doi.org/10.3389/fmars.2024.1390763

Ricciardi, A., Serrouya, R., & Whoriskey, F. G. (1995). Aerial exposure tolerance of zebra and quagga mussels (Bivalvia: *Dreissenidae*): implications for overland dispersal. *Canadian Journal of Fisheries and Aquatic Sciences*, **52**(3), 470-477.

Richards, C. S., Vera Cruz, D., Shapiro, J. W., Wootton, J. T., & Pfister, C. A. (2025). Spatially varying selection amplifies intrapopulation differentiation among phenotypic traits in the rocky-shore mussel, *Mytilus californianus*. *Ecology and Evolution*, **15**(7). https//doi.org/10.1002/ece3.71641

Ridge, J. T., Rodriguez, A. B., & Fodrie, F. J. (2017). Salt marsh and fringing oyster reef transgression in a shallow temperate estuary: Implications for restoration, conservation and blue carbon. *Estuaries and Coasts*, **40**(4), 1013-1027. https//doi.org/10.1007/s12237-016-0196-8

Román, M., Gilbert, F., Viejo, R. M., Román, S., Troncoso, J. S., Vázquez, E., & Olabarria, C. (2023). Are clam-seagrass interactions affected by heatwaves during emersion? *Marine Environmental Research*, **186**. https//doi.org/10.1016/j.marenvres.2023.105906

Román, M., Vázquez, E., Weidberg, N., Viejo, R. M., Román, S., Troncoso, J. S., Wethey, D. S., Woodin. S. A., & Olabarria, C. (2025). Legacy effects of an atmospheric heatwave are buffered by mutualistic interactions between intertidal clams and seagrass. *Frontiers in Marine Science*, **12**. https//doi.org/10.3389/fmars.2025.1706079

Román, S., Vázquez, E., Román, M., Viejo, R. M., Weidberg, N., Troncoso, J. S., Woodin, S. A., Wethey, D. S., & Olabarria, C. (2025). Understanding the effects of recent atmospheric heatwaves on seagrass-inhabited intertidal shellfish beds: a mesocosm experiment. *Marine Environmental Research*, **212**. https//doi.org/10.1016/j.marenvres.2025.107547

Rühmkorff, S., Wolf, F., Vajedsamiei, J., Barboza, F. R., Hiebenthal, C., & Pansch, C. (2023). Marine heatwaves and upwelling shape stress responses in a keystone predator. *Proceedings of the Royal Society B: Biological Sciences*, **290**(1991). https//doi.org/10.1098/rspb.2022.2262

Sadok, S., Uglow, R. F., & Haswell, S. J. (1999). Some aspects of nitrogen metabolism in Mytilus edulis: Effects of aerial exposure. *Marine biology*, **135**(2), 297-305. https//doi.org/10.1007/s002270050627

Salmond, N. H., & Wing, S. R. (2023). Sub-lethal and lethal effects of chronic and extreme multiple stressors on a critical New Zealand bivalve under hypoxia. *Marine Ecology Progress* Series, **703**, 81-93. https//doi.org/10.3354/meps14215

Santos, D., Leite, C., Pinto, J., Soares, A. M. V. M., Pereira, E., & Freitas, R. (2023). How will different scenarios of rising seawater temperature alter the response of marine species to lithium? *Science of the Total Environment*, **856**. https//doi.org/10.1016/j.scitotenv.2022.158728

Sayco, S. L. G., Cabaitan, P. C., & Kurihara, H. (2023). Bleaching reduces reproduction in the giant clam Tridacna gigas. *Marine Ecology Progress Series*, **706**, 47-56. https//doi.org/10.3354/meps14251

Sayco, S. L. G., & Kurihara, H. (2024). Bleaching and recovery in the giant clam *Tridacna crocea* from the sub-tropical waters of Okinawa. Coral Reefs, **43**(3), 773-786. https//doi.org/10.1007/s00338-024-02502-y

Scanes, E., Parker, L. M., O'Connor, W. A., Dove, M. C., & Ross, P. M. (2020). Heatwaves alter survival of the Sydney rock oyster, *Saccostrea glomerata*. *Marine Pollution Bulletin*, **158**. https//doi.org/10.1016/j.marpolbul.2020.111389

Scanes, E., Siboni, N., Rees, B., & Seymour, J. R. (2023). Acclimation in intertidal animals reduces potential pathogen load and increases survival following a heatwave. *ISCIENCE*, **26**(6). https//doi.org/10.1016/j.isci.2023.106813

Schneider, K. R. (2008). Heat stress in the intertidal: Comparing survival and growth of an invasive and native mussel under a variety of thermal conditions. *Biological Bulletin* , **215**(3), 253-264. https//doi.org/10.2307/25470709

Schneider, K. R., & Helmuth, B. (2007). Spatial variability in habitat temperature may drive patterns of selection between an invasive and native mussel species. *Marine Ecology Progress Series*, **339**, 157-167. https//doi.org/10.3354/meps339157

Schneider, K. R., Van Thiel, L. E., & Helmuth, B. (2010). Interactive effects of food availability and aerial body temperature on the survival of two intertidal *Mytilus* species. *Journal of Thermal Biology*, **35**(4), 161-166. https//doi.org/10.1016/j.jtherbio.2010.02.003

Scrosati, R. A. (2024). Recovery of intertidal mussel stands three years after the severe 2021 heatwave in British Columbia, Canada. *Diversity*, **16**(7). https//doi.org/10.3390/d16070396

Shang, Y., Chang, X., Khan, F. U., Dupont, S., Hu, M., & Wang, Y. (2025). Sex-specific trade-off under marine heat wave and food limitation: Insights from bioenergetic and intestinal microbiome in mussels. *Aquaculture*, **594**. https//doi.org/10.1016/j.aquaculture.2024.741440

Sharma, S. S., Venter, L., Frost, E. J., Alfaro, A. C., Ragg, N. L. C., & Zamora, L. N. (2024). Behavioural and physiological responses of juvenile geoduck (*Panopea zelandica*) following acute thermal stress. *Comparative Biochemistry and Physiology Part - B: Biochemistry and Molecular Biology*, **269**. https//doi.org/10.1016/j.cbpb.2023.110892

Shi, J., Kajtar, J. B., Hayashida, H., & Ugalde, S. C. (2024). Relationships between high temperatures and Pacific Oyster disease and mortality in southeast Tasmania, Australia. *Continental Shelf Research*, **273**. https//doi.org/10.1016/j.csr.2024.105173

Shi, W., Fan, X., Li, D., Chen, J., Tan, Y., Yu, X., Tian, Y., Liu, Y., Fang, L., Huo, Z., & Yan, X. (2026). Energy metabolism and the transcriptome provide new insights into the response mechanism of Manila clam (*Rudiatapes philippinarum*) between Southern and Northern China populations under high-temperature stress. *BMC Genomics*, **27**(1). https//doi.org/10.1186/s12864-026-12617-x

Shick, J. M., Widdows, J., & Gnaiger, E. (1988). Calorimetric studies of behavior, metabolism and energetics of sessile intertidal animals. *Integrative and Comparative Biology*, **28**(1), 161-181. https//doi.org/10.1093/icb/28.1.161

Siboni, N., King, W. L., Williams, N. L. R., Scanes, E., Giardina, M., Green, T. J., Ostrowski, M., O’Connor, W., Dave, M., Labbate, M., & Seymour, J. R. (2024). Increased abundance of potentially pathogenic Vibrio and a marine heatwave co-occur with a Pacific Oyster summer mortality event. *Aquaculture*, **583**. https//doi.org/10.1016/j.aquaculture.2024.740618

Slobodskova, V. V., Zhukovskaya, A. F., & Chelomin, V. P. (2012). DNA damage in the gill cells of the marine scallop *Mizuhopecten yessoensis* during anoxic stress and aerobic recovery. *Ocean Science Journal*, **47**(2), 95-100. https//doi.org/10.1007/s12601-012-0010-x

Staniek, M. A., Pansch, C., Shama, L. N. S., Mehler, K., Steinmann, A., Middelburg, J. J., & Meysick, L. (2025). Heatwave intensity drives eco-physiological responses in infaunal bivalves: A mesocosm experiment. *Limnology and Oceanography*, **70**, S417-S431. https//doi.org/10.1002/lno.70012

Stickle, W. B., Moore, M. N., & Bayne, B. L. (1985). Effects of temperature, salinity and aerial exposure on predation and lysosomal stability of the dogwhelk *Thais* (*Nucella*) *lapillus* (L.). *Journal of Experimental Marine Biology and Ecology*, **93**(3), 235-258. https//doi.org/10.1016/0022-0981(85)90242-4

Subramaniam, R. C., Cox, I., & Onyango, E. A. (2026). Participatory One Health network modelling of climate-sensitive Vibrio and antimicrobial resistance risks in the Tasmanian oyster supply chain. *Scientific Reports*, **16**(1). https//doi.org/10.1038/s41598-026-39965-8

Talevi, J., Clarke, S. B., Coffin, M. R. S., Comeau, L. A., & Filgueira, R. (2026). Physiology and behaviour of eastern oysters (*Crassostrea virginica*) and soft-shell clams (*Mya arenaria*) under hypoxic and heatwave conditions. *Marine Environmental Research*, **216**. https//doi.org/10.1016/j.marenvres.2026.107902

Talevi, J., Steeves, L., Coffin, M., Guyondet, T., Sakamaki, T., Comeau, L., & Filgueira, R. (2023). The physiological state of four commercially important bivalve species during a naturally occurring heatwave. *Canadian Journal of Zoology*, **101**(10), 913-929. https//doi.org/10.1139/cjz-2022-0215

Tam, J. C., & Scrosati, R. A. (2011). Mussel and dogwhelk distribution along the north-west Atlantic coast: Testing predictions derived from the abundant-centre model. *Journal of Biogeography*, **38**(8), 1536-1545. https//doi.org/10.1111/j.1365-2699.2011.02498.x

Tarnowska, K., Verney, A., Wolowicz, M., Feral, J. P., & Chenuil, A. (2012). Survival of male and female *Cerastoderma glaucum* (*bivalvia*) during aerial exposure. *Vie et Milieu*, **62**(1), 23-28.

Taware, S. S., Lagade, V. M., & Muley, D. V. (2012). Oxygen consumption rate of the estuarine psammobiid clam *Soletellina diphos* (linnaeus) under various environmental conditions. Indian Journal of Marine Sciences, **41**(5), 468-472.

Teaniniuraitemoana, V., Monaco, C. J., Célariès, M., Jauffrais, T., & Van Wynsberge, S. (2025). Light intensity modulates the effect of thermal stress on giant clams and their symbiotic zooxanthellae. *Coral Reefs*, **44**(5), 1449-1465. https//doi.org/10.1007/s00338-025-02708-8

Tomasetti, S. J., Hallinan, B. D., Tettelbach, S. T., Volkenborn, N., Doherty, O. W., Allam, B., & Gobler, C. J. (2023). Warming and hypoxia reduce the performance and survival of northern bay scallops (*Argopecten irradians irradians*) amid a fishery collapse. *Global Change Biology*, **29**(8), 2092-2107. https//doi.org/10.1111/gcb.16575

Torroglosa, M. E., & Giménez, J. (2019). Responses of the mussel *Brachidontes rodriguezii* to aerial exposure: Implications on growth and physiological condition. *Canadian Journal of Zoology*, **97**(7), 612-618. https//doi.org/10.1139/cjz-2018-0173

Traiger, S. B., Bodkin, J. L., Coletti, H. A., Ballachey, B., Dean, T., Esler, D., Iken, K., Konar, B., Lindeberg, M. R., Monson, D., Robinson, B., Suryan, R. M., & Weitzman, B. P. (2022). Evidence of increased mussel abundance related to the Pacific marine heatwave and sea star wasting. *Marine Ecology*, **43**(4). https//doi.org/10.1111/maec.12715

Trainer, V. L., Kudela, R. M., Hunter, M. V., Adams, N. G., & McCabe, R. M. (2020). Climate extreme seeds a new domoic acid hotspot on the US west coast. *Frontiers in Climate*, **2**. https//doi.org/10.3389/fclim.2020.571836

Trevisan, R., Mello, D. F., Le Gall, A., Corporeau, C., Fabioux, C., Huvet, A., & Paul-Pont, I. (2026). Each temperature degree counts: warming enhances polystyrene nanoplastic toxicity via metabolic disruption in a marine cellular model. *Aquatic Toxicology*, **291**. https//doi.org/10.1016/j.aquatox.2025.107685

Tucci, C. F., Milan, M., Dalla Rovere, G., Bernardini, I., Ferraresso, S., Franch, R., Babbucci, M., Monticelli, G., Panin, M., Paternello, T., Bargelloni, L., & Peruzza, L. (2025). Thermal priming mitigates the effects of lethal marine heatwaves on the Manila clam *Ruditapes philippinarum*. *ISCIENCE*, **28**(8). https//doi.org/10.1016/j.isci.2025.113108

Tucker, J. K., Theiling, C. H., Janzen, F. J., & Paukstis, G. L. (1997). Sensitivity to aerial exposure: Potential of system-wide drawdowns to manage zebra mussels in the Mississippi River. *Regulated Rivers: Research and Management*, **13**(6), 479-487. https//doi.org/10.1002/(SICI)1099-1646(199711/12)13:6<479::AID-RRR469>3.0.CO;2-B

Vajedsamiei, J., Melzner, F., Raatz, M., Kiko, R., Khosravi, M., & Pansch, C. (2021). Simultaneous recording of filtration and respiration in marine organisms in response to short-term environmental variability. *Limnology and Oceanography: Methods*, **19**(3), 196-209. https//doi.org/10.1002/lom3.10414

Vajedsamiei, J., Melzner, F., Raatz, M., Morón Lugo, S. C., & Pansch, C. (2021). Cyclic thermal fluctuations can be burden or relief for an ectotherm depending on fluctuations’ average and amplitude. *Functional Ecology*, **35**(11), 2483-2496. https//doi.org/10.1111/1365-2435.13889

Vajedsamiei, J., Wahl, M., Schmidt, A. L., Yazdanpanahan, M., & Pansch, C. (2021). The higher the needs, the lower the tolerance: Extreme events may select ectotherm recruits with lower metabolic demand and heat sensitivity. *Frontiers in Marine Science*, **8**. https//doi.org/10.3389/fmars.2021.660427

Vajedsamiei, J., Warlo, N., Meier, H. E. M., & Melzner, F. (2024). Predicting key ectotherm population mortality in response to dynamic marine heatwaves: A Bayesian-enhanced thermal tolerance landscape approach. *Functional Ecology*, **38**(9), 1875-1887. https//doi.org/10.1111/1365-2435.14620

van Erkom Schurink, C., & Griffiths, C. L. (1993). Factors affecting relative rates of growth in four South African mussel species. *Aquaculture*, **109**(3), 257-273. https//doi.org/10.1016/0044-8486(93)90168-X

Vázquez, E., Woodin, S. A., Wethey, D. S., Peteiro, L. G., & Olabarria, C. (2021). Reproduction under stress: Acute effect of low salinities and heat haves on reproductive cycle of four ecologically and commercially important bivalves. *Frontiers in Marine Science*, **8**. https//doi.org/10.3389/fmars.2021.685282

Venter, L., Alfaro, A. C., Ragg, N. L. C., Delorme, N. J., & Ericson, J. A. (2023). The effect of simulated marine heatwaves on green-lipped mussels, *Perna canaliculus*: A near-natural experimental approach. *Journal of Thermal Biology*, **117**. https//doi.org/10.1016/j.jtherbio.2023.103702

Waller, D. L., Rach, J. J., & Cope, W. G. (1995). Effects of handling and aerial exposure on the survival of unionid mussels. *Journal of Freshwater Ecology*, **10**(3), 199-207. https//doi.org/10.1080/02705060.1995.9663439

Walsh, A. R., & O'Halloran, J. (1997). The toxicity of leather tannery effluent to a population of the blue mussel *Mytilus edulis* (L.). *Ecotoxicology*, **6**(3), 137-152. https//doi.org/10.1023/A:1018614408367

Wang, J., Chuang, C.-Y., & Wang, W.-X. (2005). Metal and oxygen uptake in the green mussel *Perna viridis* under different metabolic conditions. *Environmental Toxicology and Chemistry*, **24**(10), 2657-2664. https//doi.org/10.1897/05-109R.1

Wang, T., Fan, R., Cheng, Q., Sun, Z., Fan, X., Li, N., Li, X., & Quan, W. (2020). Intertidal zonation of the Suminoe oyster *Crassostrea ariakensis* and the Kumamoto oyster *Crassostrea sikamea* on the coast of the northern East China Sea. *Journal of Shellfish research*, **39**(1), 31-41. https//doi.org/10.2983/035.039.0104

Watson, S.-A., & Neo, M. L. (2021). Conserving threatened species during rapid environmental change: Using biological responses to inform management strategies of giant clams. *Conservation Physiology*, **9**(1). https//doi.org/10.1093/conphys/coab082

Wei, S., Hu, M., Sokolova, I., Tu, Z., Chen, L., Xu, P., Mao, Y., Wang, S., & Wang, Y. (2025). Laboratory-simulated marine heatwave enhances physiological damage to mussels exposed to titanium dioxide nanoparticles by disrupting the gut−hepatopancreas axis. *Journal of Hazardous Materials*, **486**. https//doi.org/10.1016/j.jhazmat.2024.137006

Wei, S., Mao, Y., Sokolova, I. M., Li, Z., Li, L., Khalid, M. S., Tu, Z., Zhong, Z., Hu, M., & Wang, Y. (2024). Extreme heat event influences the toxic impacts of nano-TiO2 with different crystal structures in mussel *Mytilus coruscus*. *Science of the Total Environment*, **955**. https//doi.org/10.1016/j.scitotenv.2024.176916

Wei, S., Xin, Z., Lin, H., Huang, S., wen, L., Wu, L., Song, M., Wang, W., & Guo, B. (2026). Analysis of the mechanism of MKK4 participating in heat stress response in *Mytilus coruscus.* *Marine Environmental Research*, **217**. https//doi.org/10.1016/j.marenvres.2026.107956

Wei, S., Yin, Y., Tu, Z., Shang, Y., Liu, W., Xu, P., Hu, M., & Wang, Y. (2024). Repeated marine heatwaves aggravate the adverse effects of nano-TiO2 on physiological metabolism of the thick-shelled mussel *Mytilus coruscus*. *Chemosphere*, **364**. https//doi.org/10.1016/j.chemosphere.2024.143260

Wells, F. E., Keesing, J. K., Gagnon, M. M., Bessey, C., Spilsbury, F., & Irvine, T. R. (2023). Responses of intertidal invertebrates to rising sea surface temperatures in the southeastern Indian Ocean. *Frontiers in Marine Science*, **10**. https//doi.org/10.3389/fmars.2023.1075228

Wells, F. E., Keesing, J. K., Gagnon, M. M., Irvine, T. R., Bessey, C., & Spilsbury, F. (2024). Annual and decadal surveys of molluscs on intertidal platform reefs in a warming marine environment in the southeastern Indian Ocean provide a baseline for monitoring for future environmental changes. *Molluscan Research*, **44**(1), 98-106. https//doi.org/10.1080/13235818.2023.2295778

Widdows, J., & Shick, J. M. (1985). Physiological responses of *Mytilus edulis* and *Cardium edule* to aerial exposure. *Marine Biology*, **85**(3), 217-232. https//doi.org/10.1007/BF00393242

Wu, S., Wang, D., Kong, X., Zhu, X., Yang, Z., Bao, L., Bao, Z. (2026). Cardiac transcriptomic analysis of thermal stress and recovery reveals dynamic metabolic regulation in the Zhikong scallop (*Chlamys farreri*). *Aquaculture*, **613**. https//doi.org/10.1016/j.aquaculture.2025.743464

Xu, X., Tong, Y., Deng, Y., & Zhao, L. (2023). Impacts of marine heatwaves on byssus production in highly invasive fouling mussels. *Marine Environmental Research*, **184**. https//doi.org/10.1016/j.marenvres.2022.105871

Xu, X., Yang, K., Liu, Y., Deng, Y., & Zhao, L. (2023). Heatwaves hinder mussel invasion by weakening byssus production. *Frontiers in Marine Science*, **10**. https//doi.org/10.3389/fmars.2023.1239801

Xu, X., Zhang, X., Peng, J., Deng, Y., Liu, Y., Jiang, L., & Zhao, L. (2023). Survival and physiological energetics of highly invasive mussels exposed to heatwaves. *Marine Environmental Research*, **187**. https//doi.org/10.1016/j.marenvres.2023.105948

Xu, Y., Liang, J., He, G., Liu, X., Yang, K., Masanja, F., Deng, Y., & Zhao, L. (2022). Responses of pearl oysters to marine heatwaves as indicated by HSP70. *Frontiers in Marine Science*, **9**. https//doi.org/10.3389/fmars.2022.847585

Xu, Y., Luo, X., He, Y., Zang, X., Lin, W., Liu, Y., & Zhao, L. (2025). Valve gaping behavior of bivalves under compound marine heatwaves and acidification extremes. *Marine Environmental Research*, **212**. https//doi.org/10.1016/j.marenvres.2025.107557

Xu, Y., Wang, Z., Zhang, Y., Liang, J., He, G., Liu, X., Zhao, L. (2022). Transcriptome analysis reveals acclimation responses of pearl oysters to marine heatwaves. *Science of the Total Environment* **810**. https//doi.org/10.1016/j.scitotenv.2021.151189

Xu, Y., Zhang, Y., Liang, J., He, G., Liu, X., Zheng, Z., Liu, X., Zheng, Z., Deng, Y., & Zhao, L. (2021). Impacts of marine heatwaves on pearl oysters are alleviated following repeated exposure. *Marine Pollution Bulletin*, **173**. https//doi.org/10.1016/j.marpolbul.2021.112932

Yang, K., Xu, Y., He, G., Liu, X., Liu, Y., & Zhao, L. (2023). Physiological and biochemical responses of clams to recurrent marine heatwaves. *Marine Environmental Research*, **190**. https//doi.org/10.1016/j.marenvres.2023.106105

Yap, C. K., Ismail, A., & Tan, S. G. (2009). Can the tolerance of aerial exposure of the green-lipped mussel *Perna viridis* (L.) as a potential biomonitoring tool to assess Cd and Cu contamination? A laboratory study. *Asian Journal of Microbiology, Biotechnology and Environmental Sciences*, **11**(2), 263-268.

Zamora, L. N., Burritt, D. J., Ragg, N. L. C., Casanovas, P., & Delorme, N. J. (2026). Gaping restriction as a mechanism to mitigate live transport and re-immersion physiological stress in mussels. *Aquaculture*, **615**. https//doi.org/10.1016/j.aquaculture.2025.743614

Zang, X., Yang, K., Liang, H., Masanja, F., He, Y., Liu, Y., & Zhao, L. (2026). Impacts of marine heatwaves on bivalves during reproduction. *Marine Environmental Research*, **215**. https//doi.org/10.1016/j.marenvres.2025.107810

Zardi, G. I., Monsinjon, J. R., McQuaid, C. D., Seuront, L., Orostica, M., Want, A., Firth, L, B., & Nicastro, K. R. (2021). Foul-weather friends: Modelling thermal stress mitigation by symbiotic endolithic microbes in a changing environment. *Global Change Biology*, **27**(11), 2549-2560. https//doi.org/10.1111/gcb.15616

Zardi, G. I., Monsinjon, J. R., Seuront, L., Spilmont, N., McQuaid, C. D., & Nicastro, K. R. (2024). Symbiotic endolithic microbes reduce host vulnerability to an unprecedented heatwave. *Marine Environmental Research*, **199**. https//doi.org/10.1016/j.marenvres.2024.106622

Zhang, L., Zhang, Y.-Y., Ma, L.-X., & Dong, Y.-W. (2023). Evaluation of species thermal sensitivity with individual-based physiological performance. *Marine Environmental Research*, **192**. https//doi.org/10.1016/j.marenvres.2023.106212

Zhang, T., Wen, H., Xu, D., Lv, G., & Zhou, Y. (2022). PacBio full-length and illumina transcriptomes of the gill reveal the molecular response of *Corbicula fluminea* under aerial exposure. *International Journal of Molecular Sciences*, **23**(19). https//doi.org/10.3390/ijms231911474

Zhang, W.-Y., Storey, K. B., & Dong, Y.-W. (2021). Synchronization of seasonal acclimatization and short-term heat hardening improves physiological resilience in a changing climate. *Functional Ecology*, **35**(3), 686-695. https//doi.org/10.1111/1365-2435.13768

Zhang, Y., Sun, J., Mu, H., Li, J., Zhang, Y., Xu, F., Xiang, Z., Qian, P., Qui, J., & Yu, Z. (2015). Proteomic basis of stress responses in the gills of the pacific oyster *Crassostrea gigas*. *Journal of Proteome Research*, **14**(1), 304-317. https//doi.org/https//doi.org/10.1021/pr500940s

Zhao, Z., Bi, S., Zhao, L., Noor, Z., Guo, S., Wei, Z., Duan, X., Qin, Y., Ma, H., Pan, Y., Yu, Z., Shirai, K., Li, J., & Zhang, Y. (2025). Exploring the symbiotic resilience of boring giant clams and zooxanthellae during heatwave conditions: An investigation into strategic countermeasures. *Aquaculture*, 607. https//doi.org/10.1016/j.aquaculture.2025.742596

Zhilong, Z., Yimeng, C., Kaiyue, H., Dongdong, L., Zhiying, G., Xiwu, Y., Zhongming, H., &Yanjie, Q. (2025). Taurine promotes the rapid recovery of clams (*Ruditapes philippinarum*) after aerial exposure through the glutathione pathway and by inhibiting apoptosis. *Aquaculture Reports*, **42**. https//doi.org/10.1016/j.aqrep.2025.102845

Zhou, Z., Bouma, T. J., Fivash, G. S., Ysebaert, T., van IJzerloo, L., van Dalen, J., van Dam, B., & Walles, B. (2022). Thermal stress affects bioturbators' burrowing behavior: A mesocosm experiment on common cockles (*Cerastoderma edule*). *Science of the Total Environment*, **824**. https//doi.org/10.1016/j.scitotenv.2022.153621

Zhou, Z., Fivash, G. S., Cozzoli, F., Walles, B., Troost, K., Ysebaert, T., & Bouma, T. J. (2025). Compound extreme events reshuffle the stacked odds in the gamble between native and introduced bivalves. *Global Ecology and Conservation*, **64**. https//doi.org/10.1016/j.gecco.2025.e03918

Zhu, X., Zhang, J., Li, M., Hou, X., Liu, A., Dong, X., Wang, W., Xing, Q., Huang, X., Wang, S., He, J., & Bao, Z. (2023). Cardiac performance and heart gene network provide dynamic responses of bay scallop *Argopecten irradians irradians* exposure to marine heatwaves. *Science of the Total Environment*, **882**. https//doi.org/10.1016/j.scitotenv.2023.163594
